# Effect of CoSn_3_ nanocrystals on Sn3Ag plating for electronic packaging

**DOI:** 10.1038/s41598-023-48159-5

**Published:** 2023-11-24

**Authors:** Jintao Wang, Luobin Zhang, ZiWen Lv, Jianqiang Wang, Weiwei Zhang, Xinjie Wang, Hongtao Chen, Mingyu Li

**Affiliations:** 1 Department of Materials Science and Engineering, Harbin Institute of Technology (Shenzhen), Shenzhen, 518055 China; 2 State Key Lab of Advanced Brazing and Joining, Harbin Institute of Technology, Harbin, 150001 China; 3 Sauvage Laboratory for Smart Materials, Harbin Institute of Technology (Shenzhen), Shenzhen, 518055 China

**Keywords:** Materials science, Nanoscience and technology

## Abstract

Plating Sn3Ag on copper substrates represents a crucial electronic packaging technique. In this study, we propose a novel composite plating approach, wherein **CoSn**_**3**_ nanocrystals are deposited within the Sn3Ag coating. The resulting reflowed Sn3Ag joints exhibit a range of distinctive properties. Notably, **CoSn**_**3**_ nanocrystals dissolve in Sn during the reflow process, thereby lowering the supercooling required for Sn nucleation. Consequently, Sn crystals grow in six-fold cyclic twins. Additionally, the dissolution of Co atoms in Sn leads to a reduced solubility of Cu atoms in Sn, consequently lowering the supercooling required for the nucleation of Cu_6_Sn_5_. Simultaneously, this phenomenon promotes the nucleation of Cu_6_Sn_5_, resulting in a considerable precipitation of Cu_6_Sn_5_ nanoparticles within the joints. Therefore, the mechanical properties of the joints are significantly enhanced, leading to a notable 20% increase in shear strength. Furthermore, the presence and distribution of Co elements within Sn induce changes in the growth pattern of interfacial Cu_6_Sn_5_. The growth process of Cu_6_Sn_5_ is dominated by the interfacial reaction, leading to its growth in a faceted shape. During the aging process, the dissolution of Co elements in Sn impedes the continuous growth of Cu_6_Sn_5_ at the interface, causing Cu_6_Sn_5_ to be distributed in the form of islands inside the joint. Remarkably, elemental Co acts as an inhibitor for the development of Cu_3_Sn and reduces the occurrence of Kirkendall voids.

## Introduction

Composite lead-free solders containing micro-, and nanoparticles have been extensively studied^1–5^. Due to grain-boundary resistance, these particles can inhibit the coarsening of the brazing material organization, especially the **Cu**_**6**_**Sn**_**5**_, Ag3Sn intermetallic compounds and β-Sn phases. The mechanical properties of composite brazing alloys are significantly improved due to diffuse hardening or dislocation resistance. In addition, these particles affect the rate of interfacial reaction and some of them are transformed into a layer of intermetallic compounds. The wettability, creep resistance and hardness properties of the alloy braze are affected by these particles.

Srivalli et al.^6^ obtained low melting point and high hardness solder joints after addition of diamond nanoparticles (< 0.5 wt%)。Siyang Xu et al.^7^ creatively added FeCo magnetic nanoparticles (MNP) into SAC305 paste. SAC solder-FeCo MNP composite solder paste that can be locally reflowed by an AC magnetic field, and localized heating in the interconnect prevents the entire package from being subjected to the higher reflow temperatures associated with SAC solder, thereby reducing thermal stress. El-Daly et al.^8^ showed that the addition of Zn to Sn-1.0Ag-0.3Cu solder precipitated refined fibrous Ag3Sn on the surface of the β-Sn matrix and improved creep resistance and creep time. Shnava et al.^9^ determined that the addition of Fe to Sn-1.0Ag0.5Cu solder inhibited the coarsening of Ag3Sn grains without any significant effect on the melting point of the solder. The presence of Fe2NiO4 nanoparticles slightly improved the tensile properties compared to pure SAC305^10^. In addition, the presence of Fe2NiO4 nanoparticles in SAC solder hindered the growth of the intermetallic compound (IMC) layer, which reduced the average size and spacing of the IMCs .Sun et al.^11^ investigated the properties of Sn-1.0Ag-0.5Cu solder reinforced by Al nanoparticles, and found that the addition of the particles did not significantly change the melting point of the solder, but did improve its wettability and mechanical properties. The addition of a small amount of Bi to Sn-1.5Ag-0.7Cu solders reduces supercooling, refines the microstructure, reduces the nucleation rate of intermetallic compounds (IMCs), and improves creep resistance and fracture life solders. Cheng et al. reported that the addition of low levels of Ni to Sn-1.0Ag-0.5Cu solders enhanced the growth of **Cu**_**6**_**Sn**_**5**_ IMCs and inhibited the growth of Cu3Sn IMCs during solid-state aging^4^.

Presently, two widely employed methods for fabricating composite solders with added reinforcement materials are the mechanical blending method and the powder metallurgy method. In the former, solder paste and reinforcement materials are directly combined through mechanical mixing. In contrast, the latter involves mixing solder powder and reinforcement materials using ball milling, followed by compaction, sintering, and subsequent extrusion or rolling. Despite these techniques, a significant challenge arises during the soldering process: a considerable portion of the added reinforcement material is often excluded from the solder joint. Consequently, the retained ratio of reinforcement (RRoR) in the final solder joint varies substantially from the initial solder joint, leading to a diminished reinforcing effect due to the limited incorporation of the reinforcement material. It is crucial to note that the type of reinforcement, the number of reflow cycles, and the specific composite solder processing method significantly influence the RRoR in the solder joint. For a given processing method, reactive reinforcements (e.g., Ni) that interact with both solid and molten solder demonstrate a higher retention rate compared to non-reactive reinforcements (e.g., TiC) that do not react with the solder alloy. Understanding and optimizing the factors affecting the RRoR is of utmost importance to enhance the mechanical properties and performance of composite solder joints in various applications^3, 12, 13^.

**Figure 1:**
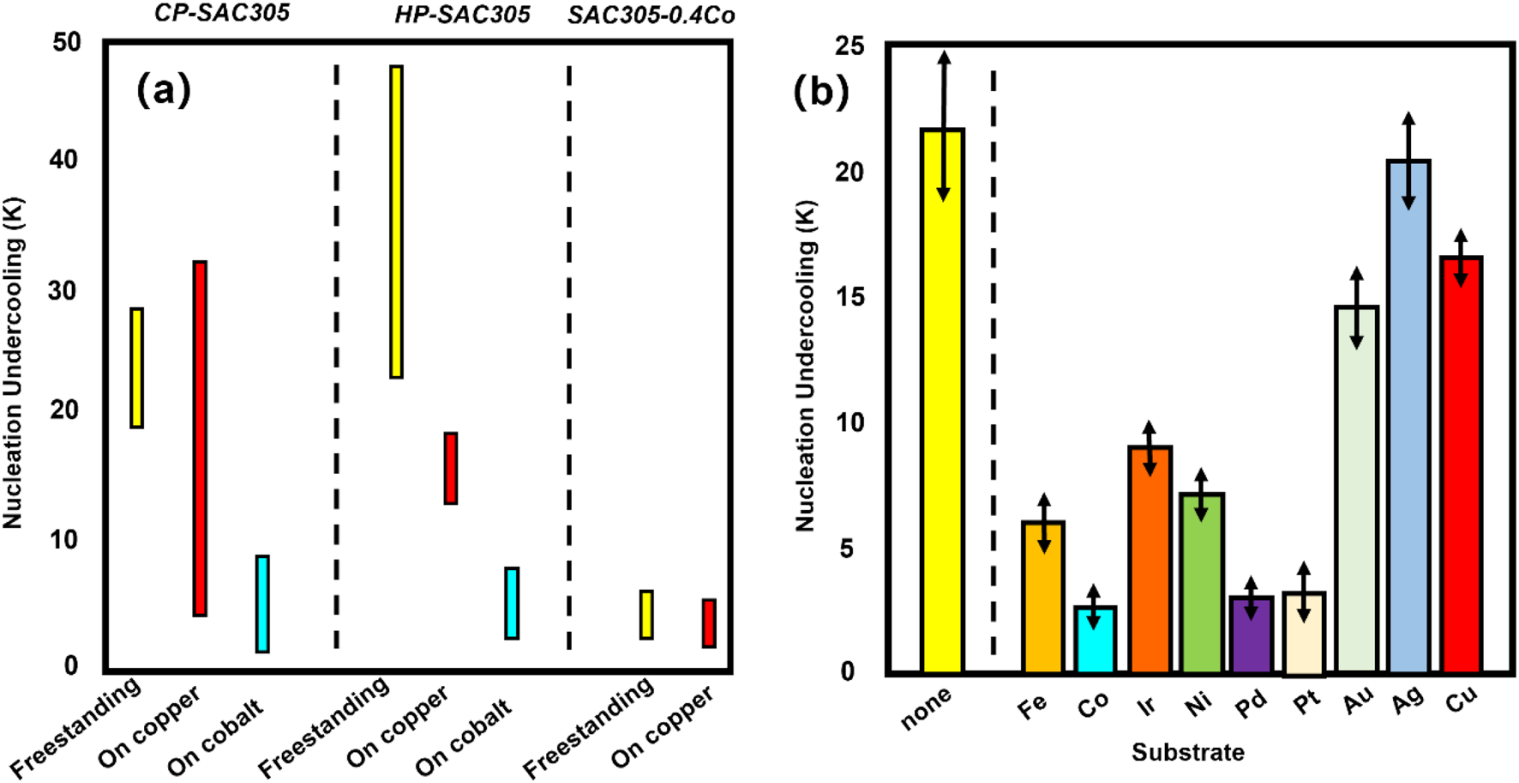
Effect of Co substrate and Co elemental doping on the supercooling required for Sn nucleation (adopt from^14^) (**a**,**b**).

Distinguishing from the above scheme, we developed a new strategy for the preparation of composite solder joints. The Sn3Ag-CoSn_3_ composite joints were prepared by composite plating. Co element is a fast-diffusing element in Sn, which helps to improve the RRoR rate, and at the same time, it can stabilize and reduce the supercooling required for Sn nucleation (Fig. 1)^14^, which will contribute to one of our new reinforcement strategies—inhibiting **Cu**_**6**_**Sn**_**5**_ nucleation and **Cu**_**6**_**Sn**_**5**_ growth by promoting **Cu**_**6**_**Sn**_**5**_ nucleation. **Cu**_**6**_**Sn**_**5**_ growth.

## Experimental methods

**Figure 2:**
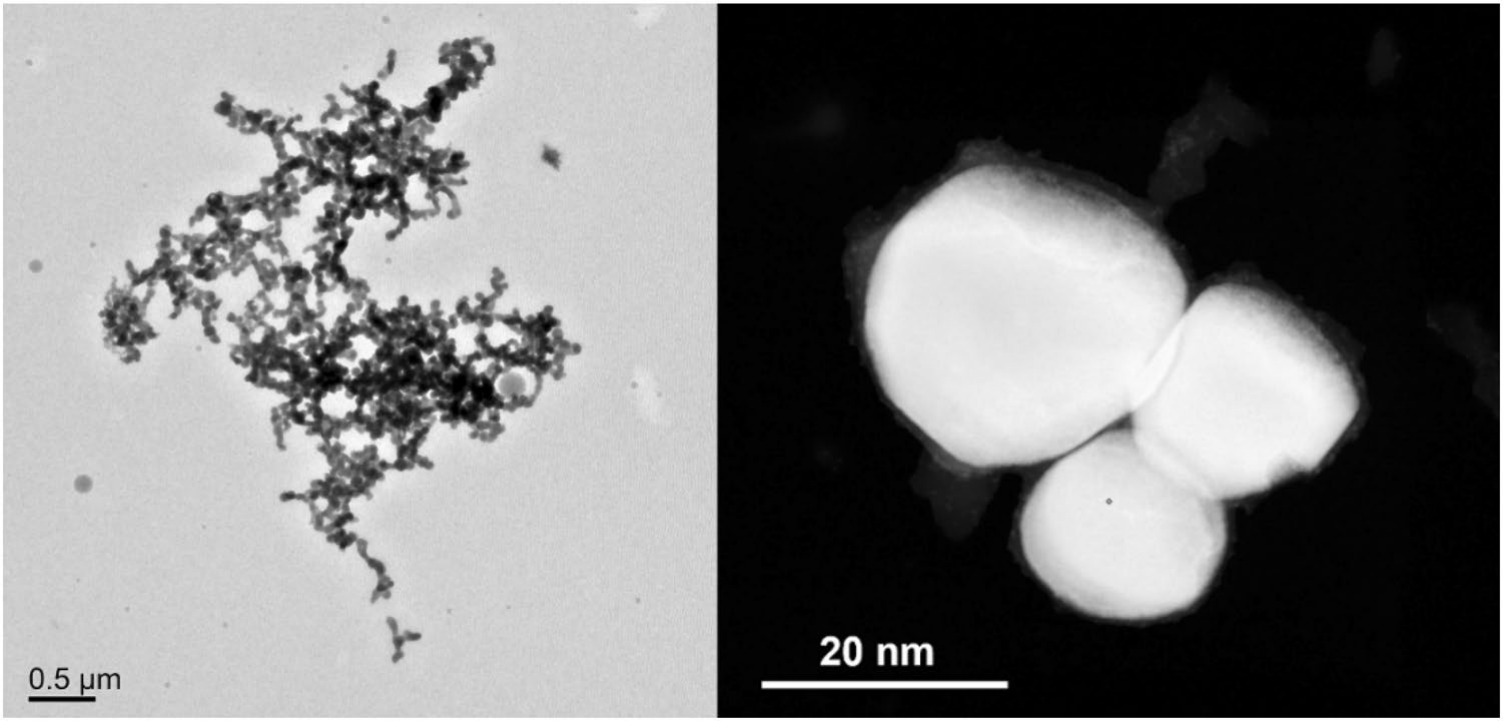
TEM images of **CoSn**_**3**_ nanoparticles.

**CoSn**_**3**_ nanoparticles (Fig. 2) were prepared by thermal synthesis and the detailed preparation method we published in^15^.

To prepare the composite plating solution, we selected a methane-sulfonic acid tin plating solution, which consisted of CH_3_SO_3_H, Sn(CH_3_SO_3_)_2_, AgCH_3_SO_3_, C_2_H_6_S_2_, and C_5_H_11_N_5_S (Based on a commercial plating solution of Sn3.0Ag with partially adapted compositions, Commercial Plating Solution by National Center for Advanced Packaging Co., Ltd.). In addition, nano CoSn_3_ particles and a dispersant, polyethylene glycol, were incorporated into the composite plating solution. By introducing cysteine into the system, we aimed to refine the grain structure by influencing the surface double layer configuration and electrode kinetics. Notably, the nanoparticles adsorb onto the high-energy surface regions, effectively inhibiting the growth of the most active Sn sites.

The relationship between the composite amount of CoSn_3_ nanoparticles in the final Sn coating and the concentration of nanoparticles in the plating solution is as follows:

$$\frac{C}{\alpha } = \frac{{Wi_{0} }}{{{\text{n}}F\eta \nu_{0} }} \cdot e^{A - B} \eta \left( {\frac{1}{K} + C} \right)$$

*C*—Concentration of nanoparticles in plating solution.

α—concentration of nanoparticles in the coating;

*ρ—*Relative density of Sn;

i_0_—Exchange current density;

*η*—Precipitation overpotential;

n,W—The relative atomic mass and valence state of Sn;

where the Sn^2+^ ion concentration was determined by titration using an iodine standard solution。Accurately remove the standard Sn^2+^ solution in a conical flask, add appropriate amount of deionized water and 1:1 hydrochloric acid solution, use a pipette to remove 1 ml of 1% starch solution, titrate with 0.1 mol L^−1^ iodine standard solution to dark blue, the end point of the color remains unchanged within 1 min, note down the volume of the iodine standard solution used at this point in time, and titrate in parallel for 3 times.

$${\varvec{\rho}}=\frac{{{\varvec{V}}}_{{\varvec{I}}}{{\varvec{C}}}_{{\varvec{I}}}}{{{\varvec{V}}}_{{\varvec{S}}{\varvec{n}}}}$$

${{\varvec{V}}}_{{\varvec{S}}{\varvec{n}}}$—Volume of Sn^2+^ solution removed.

${{\varvec{C}}}_{{\varvec{I}}}$—Concentration of standard iodine solution.

${{\varvec{V}}}_{{\varvec{I}}}$—Volume of standard iodine solution consumed.

The **CoSn**_**3**_ content and the composition of the plating were tested by an energy spectrometer (EDS) attached to a scanning electron microscope. The **CoSn**_**3**_ content was calculated by Eq. (3) using the mass fraction of Co during the test (For convenience, in this calculation we have neglected the contribution of the elements Ag and Cu to the mass):

$${\varvec{\frac{1}{\omega (Co{Sn}_{3})}=1+\left(\frac{1}{\omega (Co)}-1-\frac{3{M}_{Sn}}{{M}_{Co}}\right)\frac{{M}_{Co}}{{M}_{Co}+3{M}_{Sn}}}}$$

${\varvec{\omega (Co{Sn}_{3})}}$—Mass Ratio of **CoSn**_**3**_ in composite coatings

${\varvec{\omega (Co)}}$—Mass Ratio of Co in the results of EDS.

${\varvec{{M}_{Sn}}}$—Relative atomic mass of element Sn.

${\varvec{{M}_{Co}}}$—Relative atomic mass of element Co.

**Figure 3:**
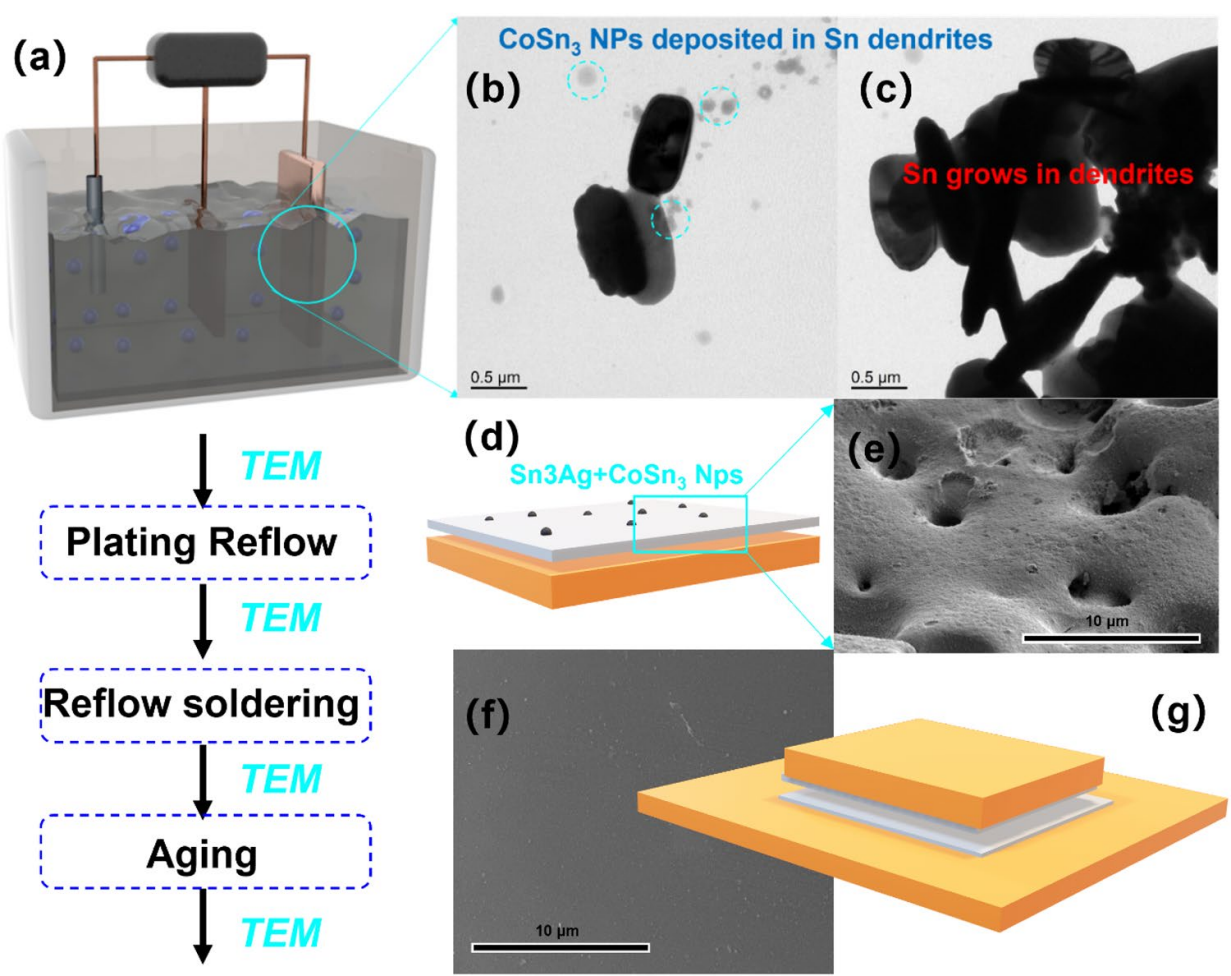
Schematic diagram of composite plating and reflow process (**a**) Composite Plating (**b**) Nucleated Sn grains and **CoSn**_**3**_ nanoparticles on copper plates. (**c**) Sn grains and **CoSn**_**3**_ nanoparticles during growth on copper plates. (**d**) Schematic diagram of composite plating (**e**) Composite plating before reflow (**f**) Composite plating after reflow (**g**) Joint reflow schematic.

Using a Cu plate as the cathode and a Sn plate as the sacrificial anode, we prepared Sn3.0Ag-plated Cu plates with CoSn_3_ mass ratios of 0%, 0.3% 0.6% 0.9% 1.2%, respectively. Plating thickness of approx. 30 microns. The SnAg plated Cu plate was reflow soldered with another SnAg plated Cu plate at 250 °C for 5 min. To facilitate analysis, the specimens were sectioned using a focused ion beam (FIB-SEM, FEI Scios 2 HiVac) system and subsequently examined under transmission electron microscopy (TEM, FEI Talos F200x G2). A creep tester (SANS, GWTA-105, 100 kg) was used to measure the shear strength of the soldered joints at room temperature at a shear rate of 0.25 mm s^−1^. The sheared sample is a 5 × 5 × 2 (mm) copper substrate soldered to a 10 × 10 × 2 (mm) copper substrate (Fig. 3).

In order to investigate the effect of CoSn_3_ nanoparticles on soldering behavior, we also prepared a series of SAC305 composite solder pastes by mechanical mixing with CoSn_3_ mass ratios of 0%, 0.3%, 0.6%, 0.9%, and 1.2%, respectively. These composite solder pastes were used for DSC experiments and wetting angle measurement experiments. Differential scanning calorimetry (DSC) curves were obtained under an N2 atmosphere, employing a heating rate of 10 °C/min (NETCH 449F5). For the wetting angle measurement experiments, the experimental temperature was 250 °C and the substrate was Cu substrate. The contact angle θ was measured by the commercial software CAST 3.0, and the measurement method was as follows: the liquid boundary was firstly identified by the CAST 3.0 software, and the boundary was fitted with a spline curve, and finally the slope of the curve at the solid–liquid contact line was calculated by the equation of the fitted curve, i.e., the contact angle. The error of the measured contact angle is ± 0.1°.

## Result and analysis

### Effect of **CoSn**_**3**_ nanoparticles on solderability

#### Wetting and reunion

**Figure 4:**
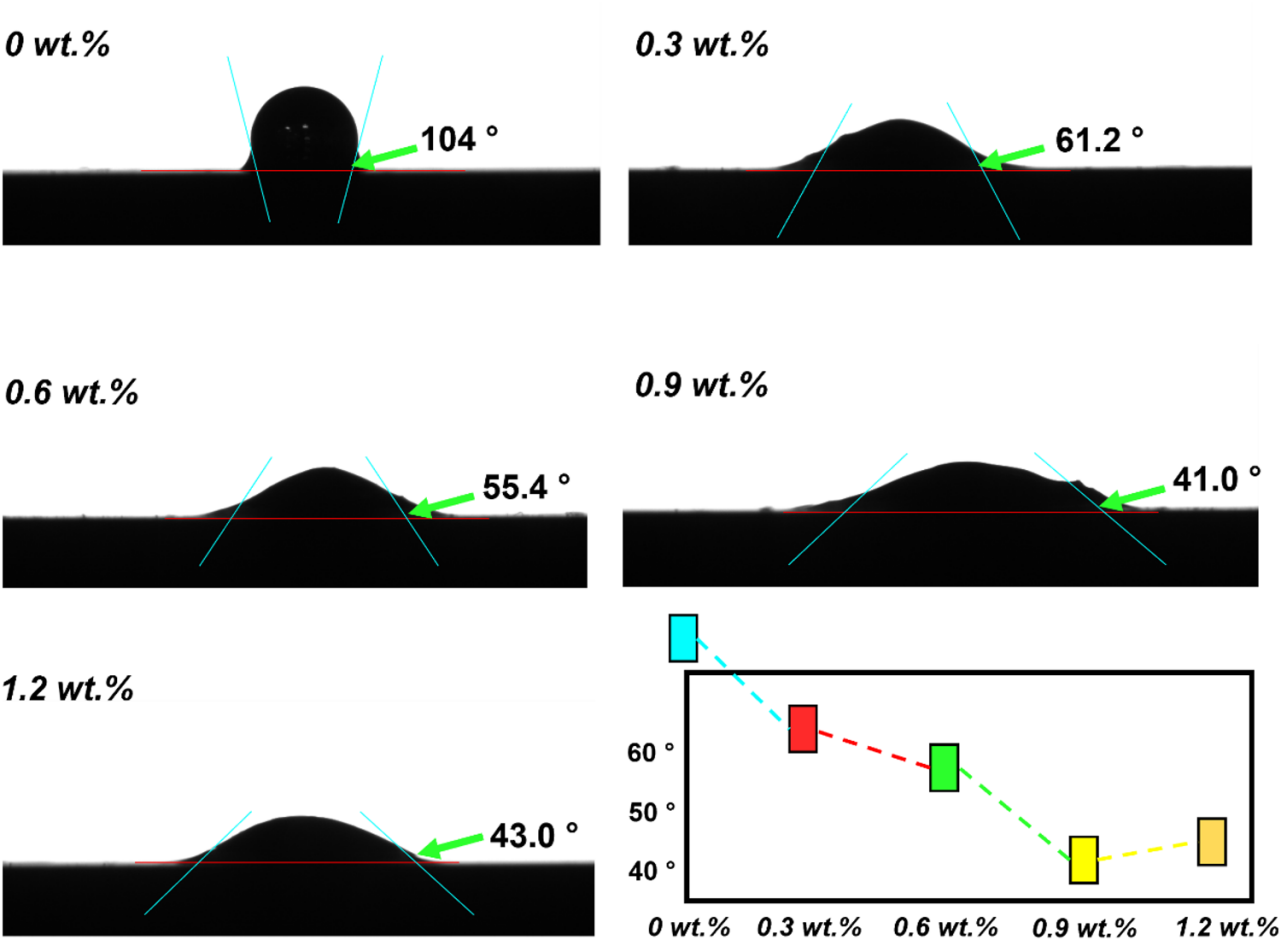
Contact angles of composite solder pastes doped with different mass ratios at 250 °C.

Nanoparticles significantly reduce the surface tension of the liquid and will enhance boiling, thus promoting early bubble departure, which helps to reduce defects and improve the quality of the bump. The microfluidic layer is the layer of liquid trapped between the heated surface and the growing bubbles. The thinner the microfluidic layer, the faster the liquid evaporates, promoting bubble growth and therefore higher heat transfer rates. In nanofluids, the orderly accumulation of nanoparticles in the curved moon face region of the microfluidic layer contributes to an increase in the extended separation pressure (i.e., fluid wettability), and the reduced surface tension also leads to an increase in the wettability of the fluid on a given solid surface. By measuring the contact angle of **CoSn**_**3**_ composite solder paste with different contents with Cu substrate at 250 °C (Fig. 4), we found that the contact angle becomes smaller rapidly with the rise of nanoparticles, but when the nanoparticles are larger than 1%, the contact angle starts to become larger again. This is due to excessive nanoparticle agglomeration.

This suggests that the addition of **CoSn**_**3**_ nanoparticles can improve the wetting performance of the solder, **CoSn**_**3**_ nanoparticles will be present in the solder alloy in a diffuse state, which makes the internal atoms in the melted molten solder alloy have weaker attraction to the surface atoms, and the liquid atom can overcome its own gravitational force more easily than that of the unadded nanoparticles and tends to the surface layer of the liquid, resulting in a better wettability performance. When the nanoparticles are adsorbed at the interface of molten solder/Cu sheet and densely distributed there, the interfacial tension ${{\varvec{\gamma}}}_{{\varvec{L}}{\varvec{S}}}$ at the interface decreases. According to Eq. (1), the decrease of interfacial tension ${{\varvec{\gamma}}}_{{\varvec{L}}{\varvec{S}}}$ increases cosθ and decreases θ, and the wetting force F increases under (Eq. 2) the condition that P, V, ${{\varvec{\gamma}}}_{{\varvec{G}}{\varvec{S}}}$, ${{\varvec{\gamma}}}_{{\varvec{L}}{\varvec{S}}}$, and ${{\varvec{\gamma}}}_{{\varvec{L}}{\varvec{F}}}$ are all unchanged; and at the same time, it promotes more rapid spreading and wetting of molten solder on the Cu sheet, thus reducing the wetting time of the composite solder. and wetting, thus reducing the wetting time of the composite solder.

However, excessive **CoSn**_**3**_ nanoparticles can agglomerate during reflow.

1 $${\varvec{c}}{\varvec{o}}{\varvec{s}}{\varvec{\theta}}=\frac{{{\varvec{\gamma}}}_{{\varvec{G}}{\varvec{S}}}-{{\varvec{\gamma}}}_{{\varvec{L}}{\varvec{S}}}}{{{\varvec{\gamma}}}_{{\varvec{G}}{\varvec{L}}}}$$

2 $${\varvec{F}}={\varvec{a}}-{\varvec{K}}{\varvec{c}}{\varvec{o}}{\varvec{s}}{\varvec{\theta}}$$

In the application of composite brazing materials, nanoparticle agglomeration poses a common challenge. The dispersion of nanoparticles belongs to a thermodynamically unstable system. Due to their substantial specific surface area, nanoparticles experience an increased proportion of atoms on their surface, resulting in insufficient atomic coordination and higher surface energy. Consequently, these particles are prone to mutual attraction, leading to the formation of larger-sized agglomerates through numerous interfacial connections. The issue of agglomeration is particularly pronounced during the soldering process, where elevated temperatures significantly promote this effect. This can be attributed to the fact that higher temperatures intensify the thermal movement (Brownian motion) of particles, leading to more frequent inter-particle collisions and, in turn, facilitating agglomeration. It is essential to address and control nanoparticle agglomeration to ensure the optimal performance and effectiveness of composite brazing materials.

However, **CoSn**_**3**_ nanoparticles have a unique feature, Co element is a fast-diffusing element in Sn, and the diffusion activation energy only needs 0.45 eV, from the phase diagram, **CoSn**_**3**_ will decompose at 345 °C, and due to the nano-size effect, **CoSn**_**3**_ nanoparticles in liquid Sn will gradually dissolve at 250 °C or higher.

### Dissolution of **CoSn**_**3**_ Ns during reflowing

**Figure 5:**
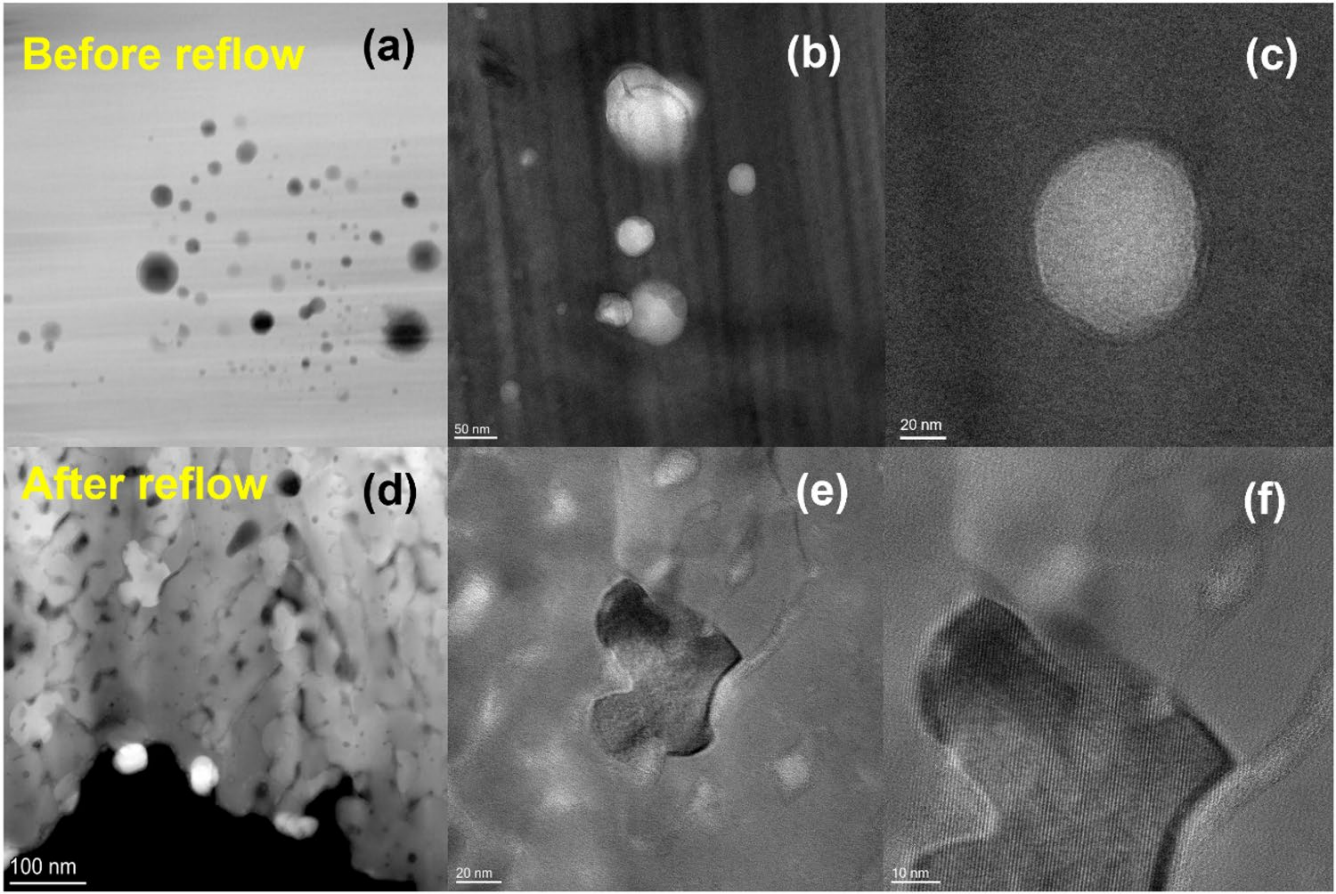
Transmission electron micrograph (TEM) of composite plating before and after reflow (**a**) **CoSn**_**3**_ nanocrystals in the plated layer (**b**) **CoSn**_**3**_ nanocrystals in the plated layer (**c**) **CoSn**_**3**_ nanocrystals in the plated layer (**d**) TEM image of composite plating after reflow (**e**) **CoSn**_**3**_ nanocrystals in the plated layer after reflow (**f**) **CoSn**_**3**_ nanocrystals in the plated layer after reflow.

**Figure 6:**
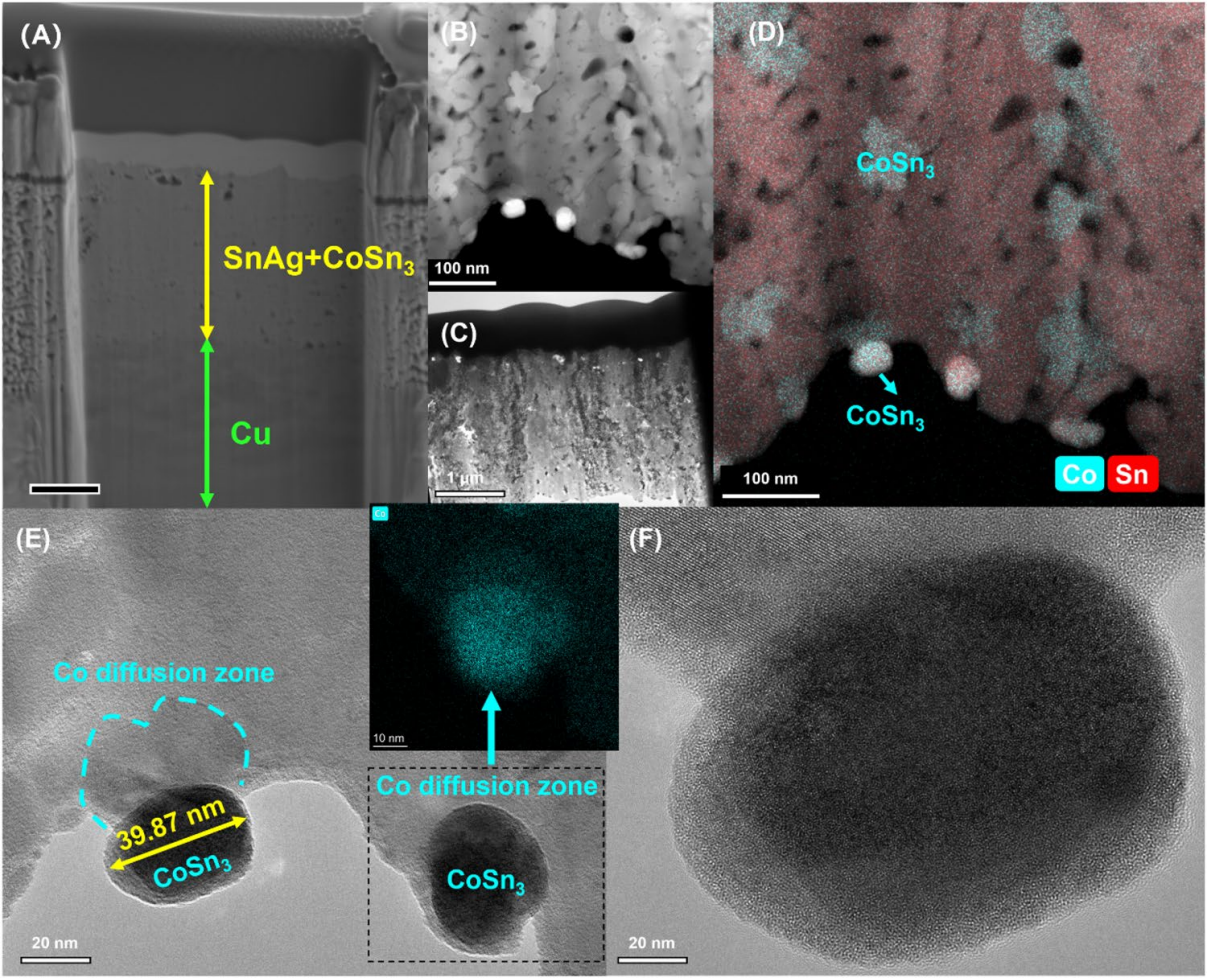
TEM images of composite plating after reflow (**A**) composite plating (**B**) composite plating (**C**) composite plating (**D**) Elemental distribution of composite plating (**E**) **CoSn**_**3**_ nanocrystals and diffusion phenomena of Co (**F**) **CoSn**_**3**_ nanocrystals.

Upon completing the plating process, we conducted reflow to achieve a flat and smooth surface. Notably, the presence of nanoparticles on the plated layer's surface caused the appearance of numerous micrometer-sized pits (Fig. 3e) after plating, which, interestingly, disappeared following reflow (Fig. 3f). Our transmission electron microscopy observations of the plated layer before and after reflow revealed intriguing changes in the morphology of intermetallic compounds. Specifically, **CoSn**_**3**_ nanocrystals exhibited rapid elemental diffusion subsequent to the reflow process (Figs. 5, 6). Concurrently, the nanoparticles underwent a transformation from their initial spherical crystalline state to a diffuse distribution pattern.

**Figure 7:**
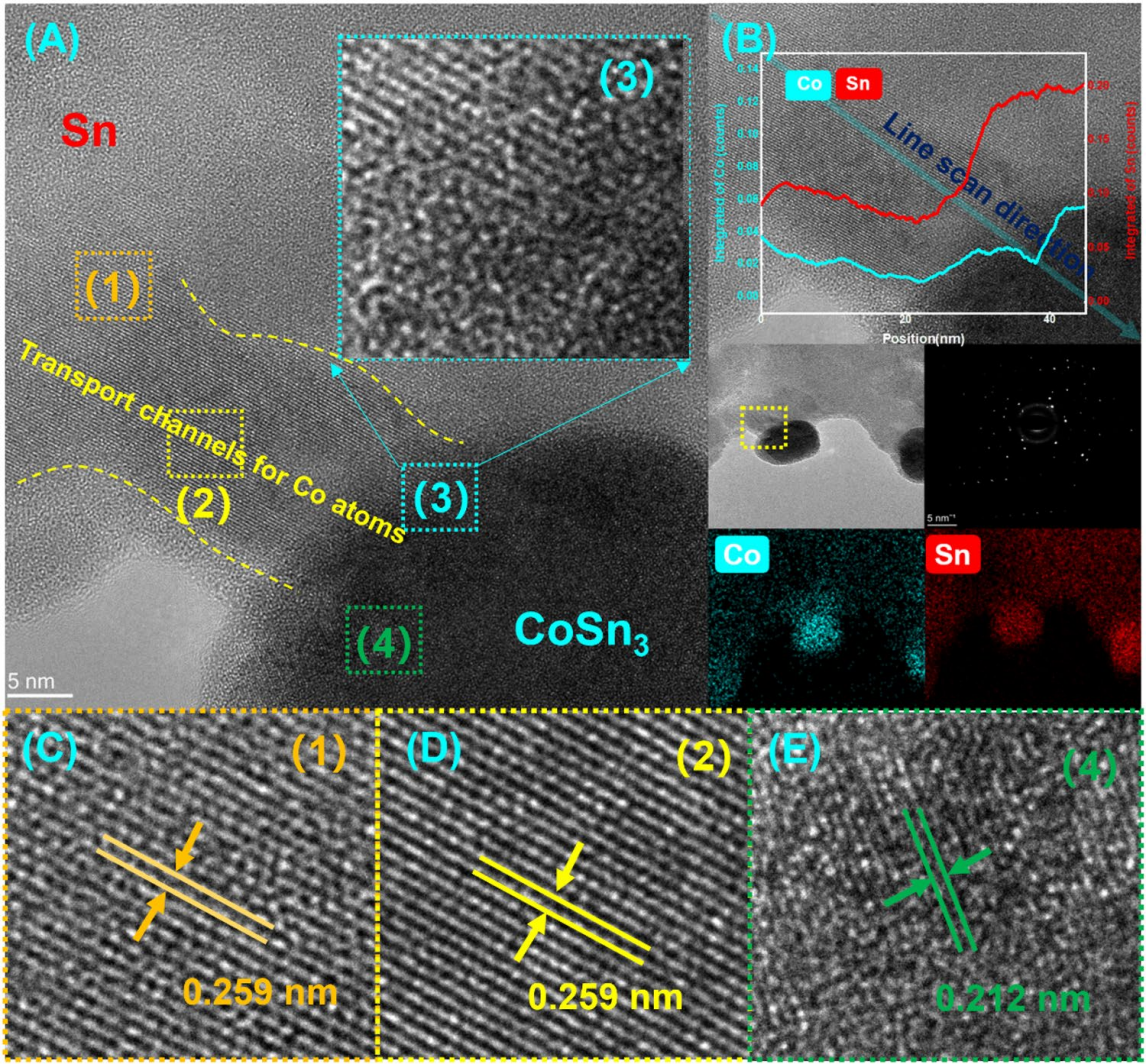
Diffusion phenomenon of Co (**A**) The emergence of ordered phases (**B**) EDX scan of the Co diffusion zone (**C**) long-range ordered structure (**D**) long-range ordered structure (**E**) Interfaces of long-range ordered structures with nanocrystals.

**Figure 8:**
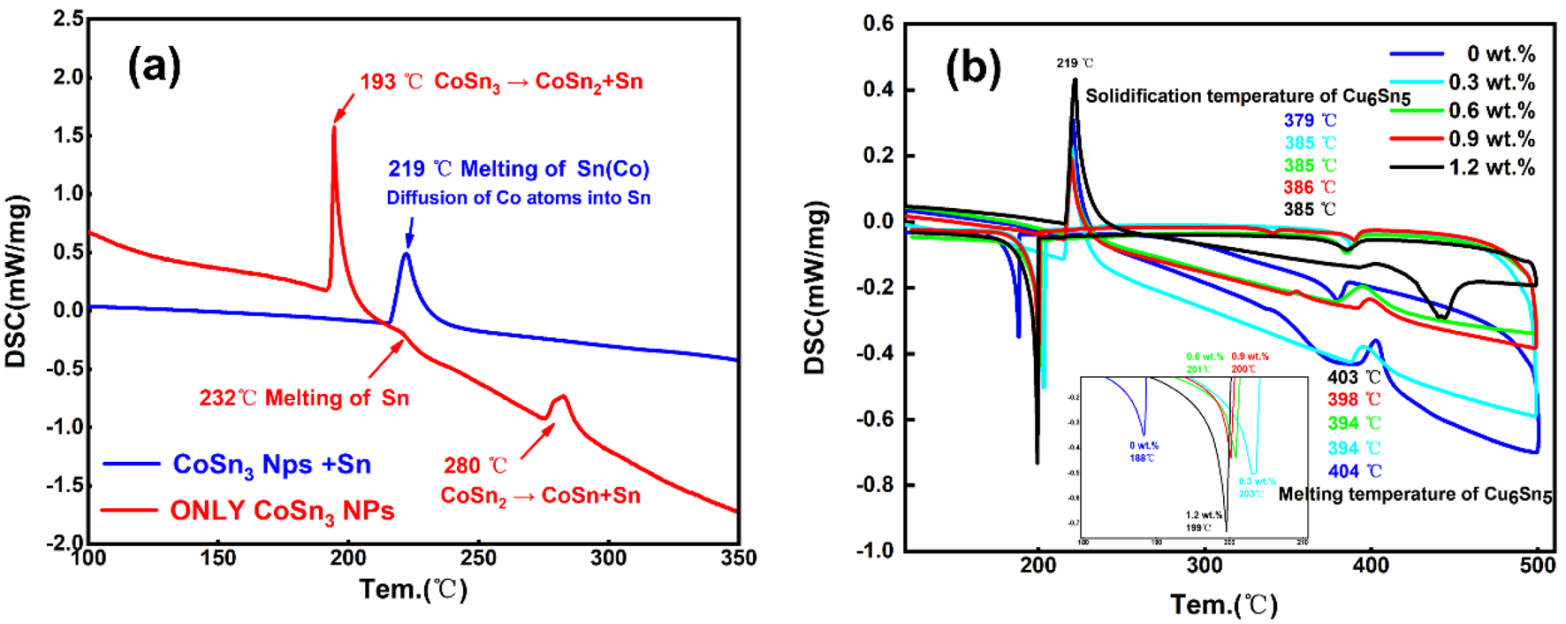
(**a**) DSC results confirmed that only CoSn_3_ nanoparticles have a decomposition peak at 193 °C and 280 °C each. However, if the CoSn_3_ nanoparticles are mixed with some Sn and heated, a heat absorption peak exists at 219 °C only. (**b**) DSC results of Composite Solder Paste (It is worth noting that, in order to ensure the diffusion of elemental Co in Sn, all the DSC data are from the second heating process, i.e., the sample was first heated to 250 °C and held for 5 min, cooled down, and then heated up to 550 °C at a rate of 10 k/min, and we performed four repetitions of the experiment, which showed only weak differences in the results of the experiment).

As we mentioned in the manuscript, a 5-min, 250 °C reflow of the plated layer is required after plating, and CoSn_3_ nanoparticles will undergo pyrolysis at about 250 °C due to surface instability, and Co atoms will leave the nanoparticles for rapid diffuse distribution into the Sn melt, and the nanoparticles will gradually disappear (Fig. 7). Our DSC experiments (Fig. 8) confirmed that simple CoSn_3_ nanoparticles have a decomposition peak at 193 °C, 280 °C each. However, if the CoSn_3_ nanoparticles are mixed with some Sn and heated, a heat absorption peak exists at 219 °C only. Due to the similar crystal structure of CoSn_3_ and Sn crystals, CoSn_3_ is extremely easy to dissolve in Sn by heating, and the Co atoms just diffuse into the whole Sn melt.

Furthermore, our observations of the nanoparticle surfaces revealed distinct diffusion channels for elemental Co, along with a gradual loss of nanoparticle surface facets and an increase in the spacing of these facets. Additionally, preferential nucleation of ordered domains was associated with specific surface facets, leading to the establishment of a fully ordered phase (Fig. 7), eventually culminating in pre-melting. The dynamic coupling of surface facets and elemental interdiffusion during reflow was driven by a decrease in free energy resulting from negative surface polarization enthalpies. This decrease induced surface compositional ordering, leading to a subsequent decrease in the system's conformational entropy. Nevertheless, it should be noted that the surface polarization free energy eventually became positive as a result of the increased entropy contribution. Importantly, thermally activated elemental diffusion was found to be temperature-dependent, while the duration of reflow played a significant role in enabling efficient atomic motions^16^.

As a result, we found that a large amount of liquid-phase Sn separated from CoSn_3_ after heating at 250 °C for 5 min. In a very short time afterwards, CoSn_2_ also dissolved in liquid Sn. Interestingly, during this process, CoSn_3_ did not dissolve directly, but degraded into CoSn_2_ and Sn, followed by diffusion of Co atoms in the liquid Sn. This process proves that the conversion of CoSn_3_ to CoSn_2_ is reversible. The energy input during this process selectively destroys the extruded tin lattice, releasing the tin atoms. CoSn_2_ does not continue to degrade, but dissolves directly in liquid Sn.

It is worth noting that this structural change is not entirely surprising. The crystal structure of CoSn_3_ is less stable than that of CoSn_2_. The degradation process of CoSn_3_ to CoSn_2_ and Sn is attributed to the release of excess energy to the surrounding environment. The energy released during the process of converting CoSn_3_ to CoSn_2_ is used to destroy the squeezed Sn lattice and release Sn atoms. This process confirms the metastability of CoSn_3_ in the presence of liquid phase Sn. That is to say, the ASB mechanism is reversible. We have used this feature in the field of electronic packaging by co-deposition to create CoSn_3_ Cu pillar bumps. After reflow at 250 °C, Co diffuses from the CoSn_3_ nanocrystals across the interface and the CoSn_3_ nanoparticles disappear and are replaced by Cu6Sn5 nanoparticles (the Co element reduces the Cu6Sn5 nucleation and promotes Cu6Sn5 nucleation).

According to the Co-Sn phase diagram, the **CoSn**_**3**_ phase decomposes at 345 °C. However, when **CoSn**_**3**_ exists in a nano-sized form, gradual dissolve occurs at around 250 °C in the presence of liquid Sn (Fig. 8-a). During this process, the spacing between Sn atoms in the liquid Sn environment increases, providing favorable conditions for the diffusion of Co elements. Consequently, the spherical structure of **CoSn**_**3**_ is lost, and coupling with Sn at the interface takes place. This coupling effect impedes the collision and aggregation of **CoSn**_**3**_ particles under the influence of heat. As the reflow time is extended, the Co element becomes uniformly distributed throughout the Sn interface. This uniform distribution facilitates the subsequent nucleation behavior of Sn with Cu_6_Sn_5_. The well-distributed Co element enhances the nucleation process, contributing to improved overall performance and reliability in soldering applications.

### The effect of **CoSn**_**3**_ on the microstructure

#### The effect of **CoSn**_**3**_ on Sn crystals

The results obtained from the DSC experiments demonstrate that the diffusion of Co significantly reduces the degree of subcooling required for Sn nucleation. Specifically, the incorporation of 0.3 wt% **CoSn**_**3**_ nanoparticles reduces the subcooling needed for Sn nucleation by 20 K (Fig. 8). However, it is observed that an excessive amount of **CoSn**_**3**_ nanoparticles does not yield a notably superior effect, although the difference is not substantial. This phenomenon is attributed to a large quantity of Co being involved in the Cu_6_Sn_5_ nucleation during reflow, leading to only a minor variation in the overall outcome. Further exploration of the impact of Co on Cu_6_Sn_5_ nucleation will be undertaken in the subsequent section.

In near-eutectic SnAg samples, the solidification process plays a pivotal role in determining the morphology and orientation of Sn grains. The solidification temperature influences the size of the Sn dendrite arms and the magnitude and number of precipitates. Additionally, it significantly affects the concentration of solidified Sn(Ag) liquid, as well as the size, quantity, and orientation of Sn grains. These factors collectively contribute to the overall microstructure and properties of the near-eutectic SnAg alloy. The investigation of the solidification process is critical for comprehending the underlying mechanisms that dictate grain formation and growth, shedding light on the fundamental factors influencing the material's performance and behavior^17^.

Sixfold cyclic growth twinning of Sn commonly occurs upon the solidification of SnAg, SnCu and SnAgCu melts. Cyclic twinning is not generally observed in pure Sn^18^. This particular type of twinning is common in tetragonal and orthorhombic minerals, but is less frequently observed in metal systems^17, 19^. That is to say that SnAg eutectic will all change to six-fold cyclic twinning when sufficiently heated and sufficiently supercooled (supercooling greater than 80 °C is required). However, in the usual electronic packaging process, where the reflow time is short and the reflow temperature is low to avoid harming the chip, the crystals grow very fast in SnAg solder systems by dendrite growth within the melt. Therefore, SnAg systems do not produce faceted crystals. When the critical solid nucleus forms, it grows so fast that the solder warms significantly during solidification, inhibiting additional sources of nucleation. As a result of this reflection, only the first nucleus has the best chance of growth.

**Figure 9:**
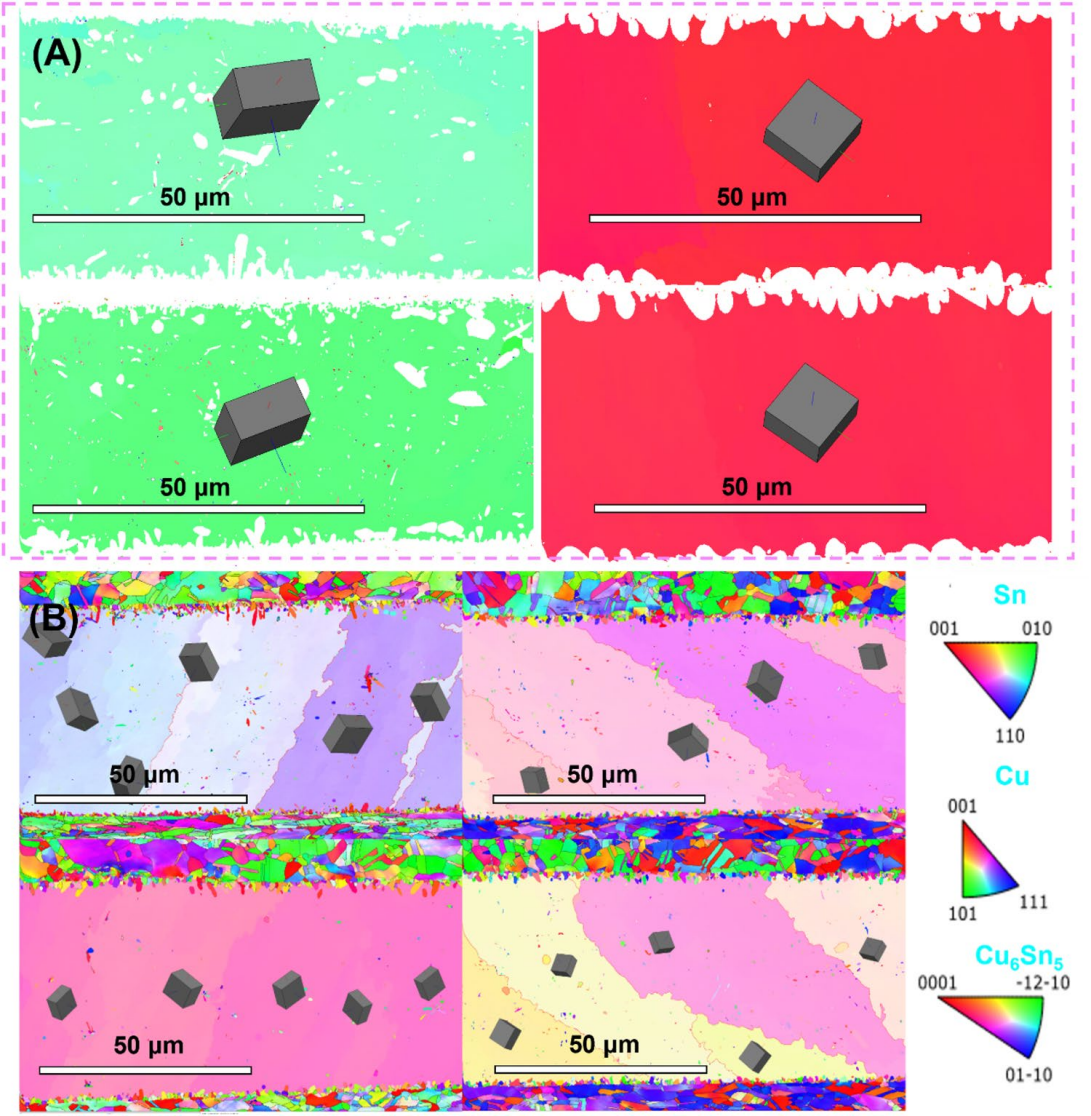
(**A**) EBSD results of solder joint without **CoSn**_**3**_ Ns (**B**) EBSD results of solder joint with 0.3 wt% **CoSn**_**3**_ Ns.

From the electron backscatter diffraction (EBSD) results (Fig. 9), we noted a striking difference between samples with and without added **CoSn**_**3**_ nanoparticles. Samples lacking **CoSn**_**3**_ nanoparticles did not exhibit six-fold cyclic twinning, while those containing 1 wt% **CoSn**_**3**_ nanoparticles displayed six-fold cyclic twinning. The diffusion of Co not only reduces the degree of supercooling required for Sn nucleation but also promotes the formation of six-fold cyclic twinning in the SnAg eutectic at a lower degree of supercooling. Typically, in Sn-based solders, twinning occurs on one of two distinct crystal planes, namely, {1 0 1} and {3 0 1} planes. In our experimental observations, cyclic twinning exclusively took place in the {101} plane, forming the basis of our subsequent discussions.

The mechanical response of cyclic twins can be advantageous for SnAg eutectic joints, as all crystal segments within a cyclic twin share a common crystal axis. Consequently, this group behaves as single crystals along the twin axis and as polycrystals in other directions, thus favoring the overall mechanical properties of SnAg eutectic joints. The EBSD results typically reveal the presence of only one set of six Sn twins in a given SnAg sample, which aligns with the occurrence of multiple twinning events associated with the nucleation process. While the morphology of Sn grains may differ across samples, the orientation of the Sn grains consistently indicates a specific type of hexagonal twin nucleation. This observation aligns with findings from previous studies.^7, 17^. In order to fill the space, the average value of all twinned sections must be 60°. Mismatches are required at the boundaries between the sections, or low angle grain boundary systems are required inside the grains between the twin interfaces.

In growth, the slow-growth^18^ direction aligns towards the neighboring twin and remains perpendicular to the fast-growth direction. This specific orientation ensures minimal competition between adjacent twins. Secondary dendrites extending from the primary [0 0 1] branches fill the gaps between the rapidly growing twins, leading to some interpenetration of the secondary dendrites of neighboring twins, resulting in irregular interfaces, as commonly observed (Fig. 9B). The occurrence of hexagonal cyclic twinning events is associated with the nucleation of Sn in substile/pseudohexagonal structures. Nucleation events can be promoted by impurities such as Cu or Ag, with Co having a particularly pronounced effect. However, this hexagonal crystal structure becomes unstable once it grows, which accounts for the observation of irregular interfaces. Interestingly, body-centered tetragonal Sn cells can grow epitaxially on hexagonal nuclei, adopting a relatively low-energy configuration as the twin interface possesses lower energy than the other cells. This phenomenon helps explain why the addition of Co significantly reduces the supercooling required for Sn nucleation. The presence of Co favors the formation of a more stable and energy-efficient twin interface during the nucleation process.

#### The effect of **CoSn**_**3**_ on Cu_6_Sn_5_ IMC

**Figure 10:**
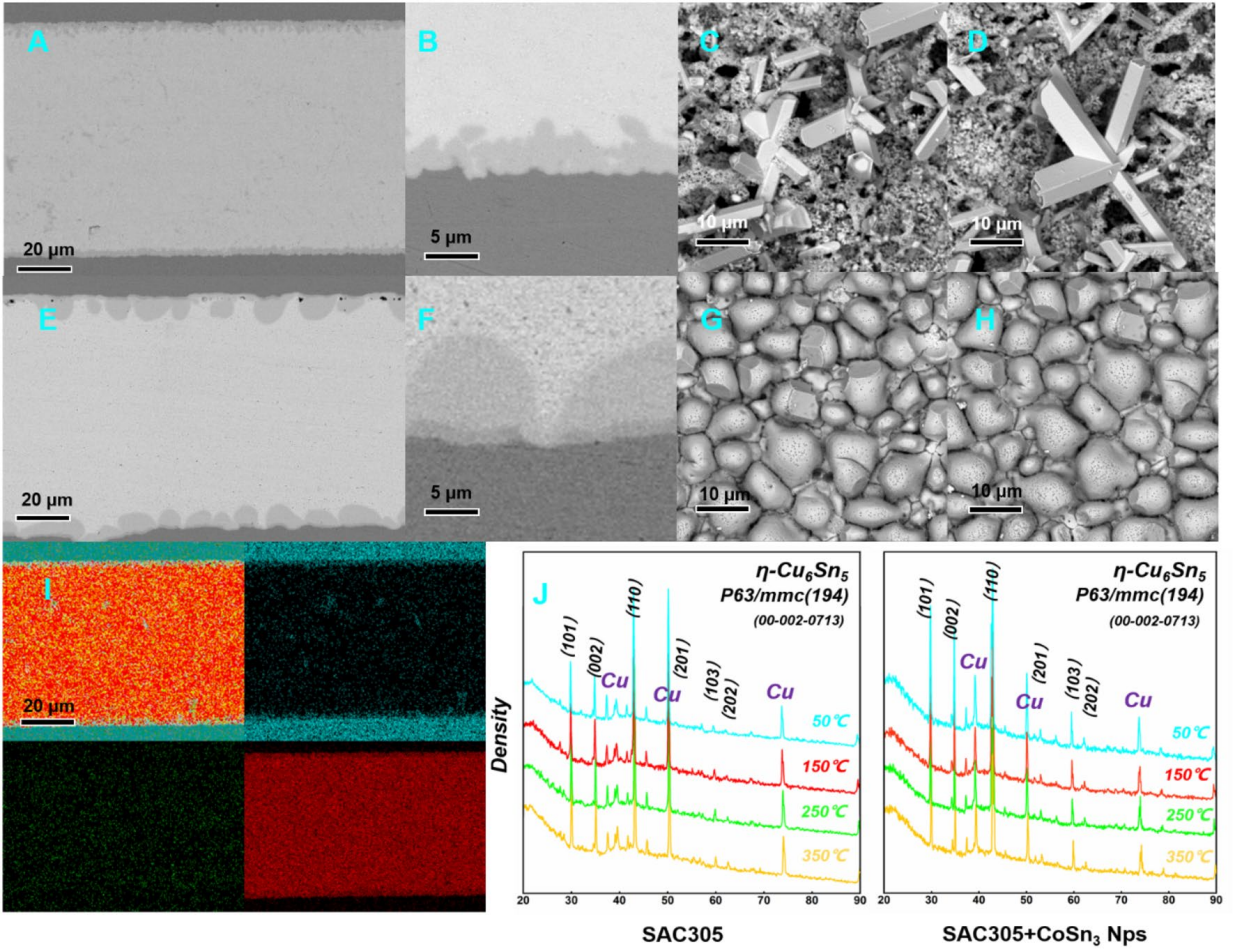
SEM images of solder joint after reflow (**A**) solder joint with 0.3 wt% **CoSn**_**3**_ Ns(**B**) interface of the solder joint with 0.3 wt% **CoSn**_**3**_ Ns (**C**) Faceted Cu_6_Sn_5_ IMC shape (**D**) Faceted Cu_6_Sn_5_ IMC shape (**E**) solder joint without **CoSn**_**3**_ Ns (**F**) interface of the solder joint without (**G**,**H**) Scalloped Cu_6_Sn_5_ (**I**) EDX mapping of solder joint with/without **CoSn**_**3**_ Ns (**J**) XRD results of solder joint with/without **CoSn**_**3**_ Ns.

The influence of **CoSn**_**3**_ on the nucleation and growth behavior of Cu_6_Sn_5_ is considerably more pronounced. Notably, the morphology of the intermetallic compound (IMC) formed by SA-Co/Cu exhibits distinct differences from the typical scalloped Cu_6_Sn_5_ formed between Sn-3Ag and Cu. The IMC morphology tends to be more intricate, and the unique two-phase structure of the IMC becomes more evident. Specifically, in the SA-Co/Cu interface, an increased number of Sn-rich "island" phases were observed in the IMC region. A comprehensive top-view analysis was conducted to reveal the integrated morphological evolution of the IMC, as depicted in Fig. 10. It is evident that the IMC morphology is non-uniform, wherein the outer region of the IMC exhibits an elongated and multifaceted shape. Figure 5 provides a magnified view of the IMC grains in the external region, clearly demonstrating the formation of prismatic shapes.

The addition of **CoSn**_**3**_ significantly impacts the microstructure of the IMC, leading to novel and complex morphological characteristics. The observed prismatic shapes and the presence of Sn-rich "island" phases in the IMC region indicate the intricate nature of the interfacial reactions involving SA-Co/Cu.

One view is that the formation of IMC morphology is highly dependent on the enthalpy change during the interfacial reaction between solder and substrate. Therefore, it can be inferred that cobalt's participation in the interfacial reaction process changes the enthalpy change, which in turn affects the Jackson parameter^20^. The Jackson parameter is an indicator of the degree of atomic adhesion to the growth interface, and if the Jackson parameter is greater than 2, the IMC at the solder/copper interface will produce a multi-faceted shape, and on the contrary, a rounded IMC will be formed when the Jackson parameter is less than 2. The Jackson parameter α is defined as follows^21, 22^:

$$\boldsymbol{\alpha }=\frac{\Delta {\varvec{H}}}{{\varvec{R}}{{\varvec{T}}}_{{\varvec{s}}{\varvec{o}}{\varvec{l}}{\varvec{d}}{\varvec{e}}{\varvec{r}}{\varvec{i}}{\varvec{n}}{\varvec{g}}}}{\varvec{\xi}}$$

**Figure 11:**
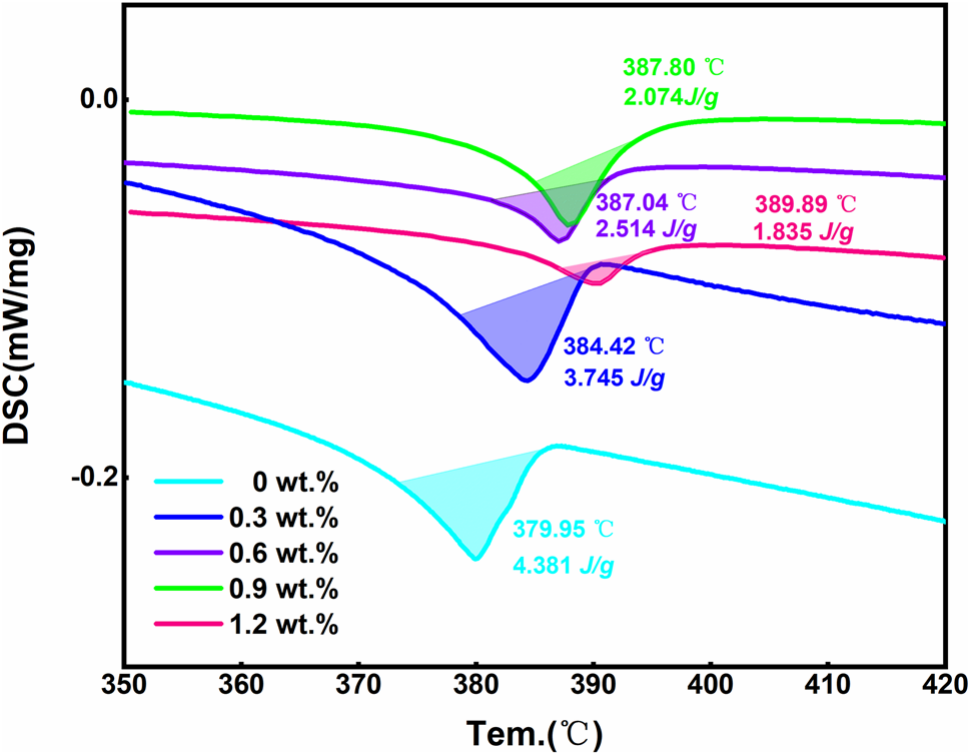
DSC results of the enthalpy change of Cu_6_Sn_5_ nucleation.

$\Delta {\varvec{H}}$ denotes the enthalpy change during the interfacial reaction, R is the universal gas constant, ${\varvec{\xi}}$ represents the fraction of the total number of nearest neighbors in a plane parallel to the interface under consideration. ${{\varvec{T}}}_{{\varvec{s}}{\varvec{o}}{\varvec{l}}{\varvec{d}}{\varvec{e}}{\varvec{r}}{\varvec{i}}{\varvec{n}}{\varvec{g}}}$ is the soldering temperature. That is, as $\Delta {\varvec{H}}$ gets larger, IMC is more likely to form faceted shape. We measured the enthalpy change of the reaction of Cu with Sn to form Cu_6_Sn_5_ at different Co contents by DSC, see Fig. 11.

From the DSC results (Fig. 11), Co doping reduces the degree of subcooling required for the formation of Cu_6_Sn_5_ and reduces the enthalpy change of the reaction. Therefore, the Jackson parameter does not increase and the above theory cannot explain the change in the morphology of Cu_6_Sn_5_ IMC (Co doping makes the $\Delta {\varvec{H}}$ smaller). In other words, the Jackson parameter is not increased (but decreased), but the morphology of the intermetallic compound is changed (from scalloped to faceted). According to the conventional theory, the intermetallic compound morphology changes only when the Jackson parameter increases, which is contradictory to the experimental phenomenon, so the conventional theory cannot explain the reason for the change in the morphology of Cu_6_Sn_5_ under the influence of Co atoms. In addition, from the XRD results, the doping of Co element does not affect the crystal structure of Cu_6_Sn_5_, and the dot lattice of Cu_6_Sn_5_ is still hexagonal crystal system (P63/mmc).

**Figure 12:**
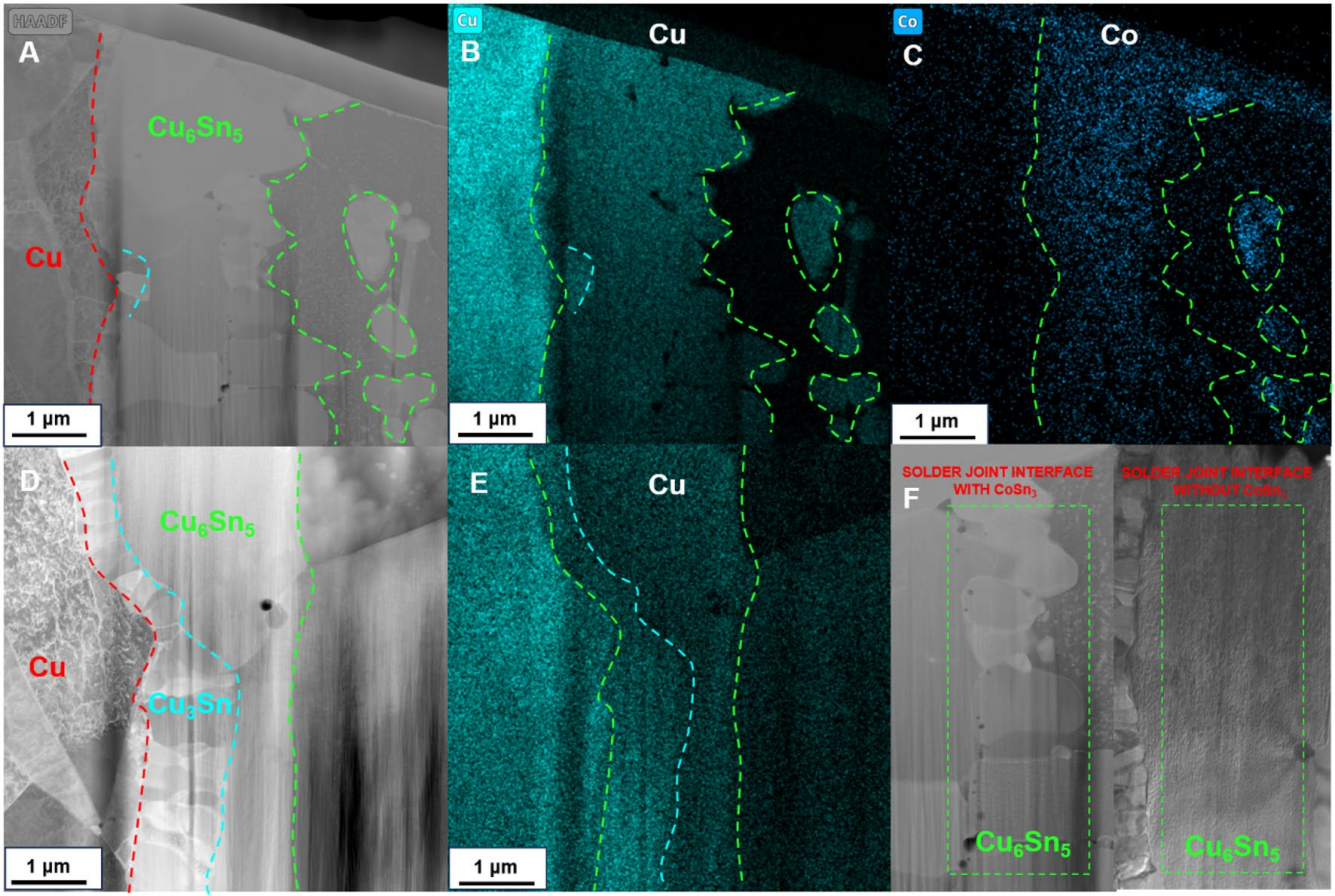
(**A**) TEM images of interface for solder joint with 0.3 wt% **CoSn**_**3**_. (**B**,**C**) Highly overlapping distribution of Cu and Co elements (**D**) TEM images of interface for solder joint without **CoSn**_**3**_ Ns (**E**) Distribution of Cu elements on the connector without **CoSn**_**3**_ (**F**) Comparison of Cu_6_Sn_5_ grain size, the left side is the joint with 0.3 wt%**CoSn**_**3**_ of doping, the right side is the joint without doping.

From the EDX results (Fig. 12), highly overlapping distribution of Co and Cu elements, and the outer area of the (Cu, Co)6Sn5 region on the solder side contained much higher Co additive concentration than the inner area on the Cu side. In the previous section we have explained that **CoSn**_**3**_ pyrolyzes and releases Co atoms during reflowing. This indicates that the Co element is involved in the Cu-Sn reaction and replaces some of the Cu elements:

$$6\left(1-{\varvec{x}}\right){\varvec{C}}{\varvec{u}}+5{\varvec{S}}{\varvec{n}}+6{\varvec{x}}{\varvec{C}}{\varvec{o}}\to {\left({{\varvec{C}}{\varvec{u}}}_{1-{\varvec{x}}}{{\varvec{C}}{\varvec{o}}}_{{\varvec{x}}}\right)}_{6}{{\varvec{S}}{\varvec{n}}}_{5}+\Delta {\varvec{H}}$$

**Figure 13:**
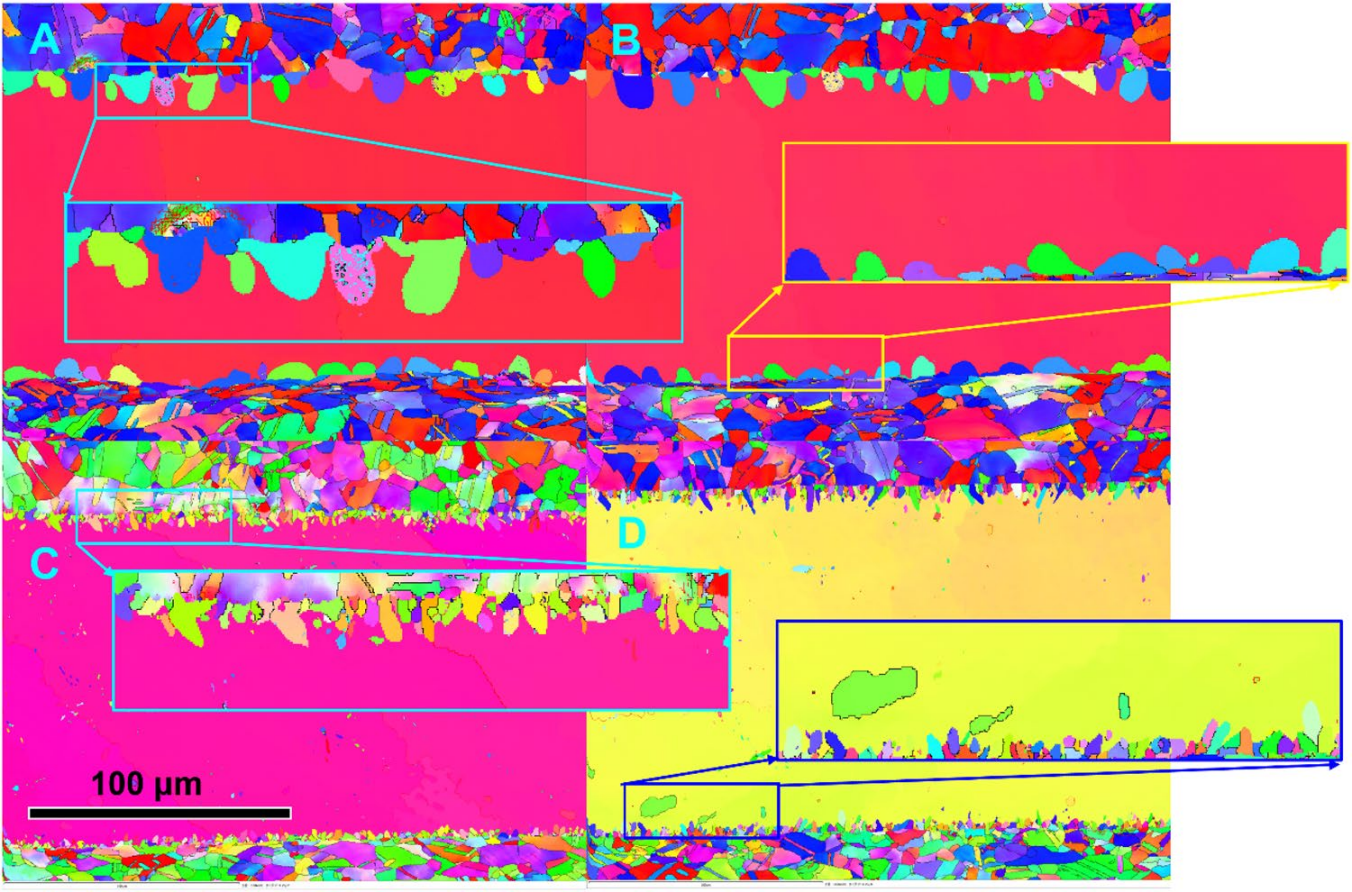
EBSD results of Cu6Sn5 in solder joint (**A**,**B**) interface of the solder joint without **CoSn**_**3**_ (**C**,**D**) interface of the solder joint with 0.3wt.% **CoSn**_**3**_ Ns.

A comparison between the grain size of Cu_6_Sn_5_ at the Cu/Sn interface of **CoSn**_**3**_-doped joints and that of Cu_6_Sn_5_ at the interface of Cu/Sn joints without **CoSn**_**3**_ doping (Figs. 12F, 13) reveals that the grains of Cu_6_Sn_5_ in the **CoSn**_**3**_-doped joints are finer. The presence of Co in the Sn reduces the solubility of Cu in Sn, effectively lowering the activation energy required for the nucleation of Cu_6_Sn_5_. This leads to an increased number of nucleation centers for Cu_6_Sn_5_, resulting in the refinement of Cu_6_Sn_5_ grains. Moreover, the rapid diffusion of Cu into Sn further facilitates an increase in the nucleation events of Cu_6_Sn_5_. Notably, the diffusion of Cu atoms is primarily governed by diffusion along the grain boundaries rather than bulk diffusion.

Concerning the growth of Cu_6_Sn_5_, two distinct behaviors are observed based on the dominant diffusion mechanism. Cu_6_Sn_5_ dominated by bulk diffusion tends to grow in a scallop shape, while Cu_6_Sn_5_ controlled by grain boundary diffusion tends to grow with facet shapes. The preference for facet shapes arises from the fact that Cu_6_Sn_5_ growth is primarily driven by Cu elemental diffusion. Consequently, Cu_6_Sn_5_ grows significantly faster in one direction compared to others, with these directions corresponding to the non-dense rows of crystal directions in that plane. As a result, the prismatic part elongates rapidly due to faster crystal growth along these directions. These observations offer valuable insights into the crystallographic principles governing the growth behavior of Cu_6_Sn_5_, highlighting the role of **CoSn**_**3**_ doping in influencing the microstructure and morphology of intermetallic compounds in solder joints.

Conventional theory^21, 22^ suggests that whether the shape of a solid–liquid interface is planar or circular depends on the energy of the chemical bonds. If the interface is planar, the adsorbed atoms on the surface of the solid phase tend to fill the first layer before filling the next layer. This results in a small number of junctions at the interface when viewed at the atomic scale. If the interface is circular, the interface is rough and uneven at the atomic scale, and there are many junctions. Therefore, there must be many steps and knots on a circular interface. Since the steps and junctions have many broken chemical bonds, the solid–liquid interface will have more steps and junctions when the bond breaking energy is low. The bond breaking energy is related to the surface energy of the solid–liquid interface, and when the surface energy is low, the bond breaking energy is also low, but it does not seem to apply to our present situation (not supported by the DSC results).

**Figure 14:**
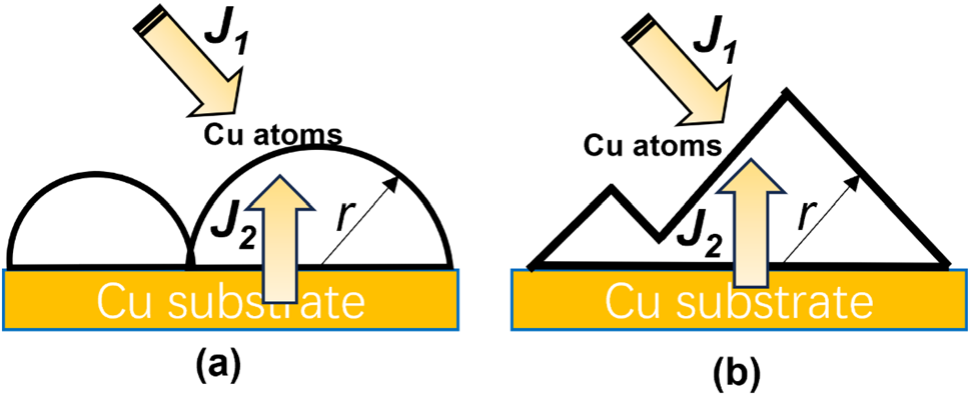
Schematic diagram of the growth mechanism of Cu_6_Sn_5_ (**a**) Scalloped (**b**) Faceted.

So we consider a suitable interpretation from the Gibbs–Thomson principle^23–25^. Gibbs–Thomson principle allows a relationship to be established between the curvature of the solid–liquid interface and the equilibrium concentration of solute atoms near the interface, which has important applications to the study of growth control and aging problems of intermetallic compounds. The growth of intermetallic compounds mainly originates from two kinetic processes, i.e., interfacial reaction process (J_1_) and aging action (J_2_). Therefore, the equilibrium relationship between the fluxes of these two actions determines the growth kinetics of intermetallic compounds (Fig. 14).

Assume that the intermetallic compound grain is a hemispherical grain with radius r. The concentration of Cu atoms in the molten Sn on the surface of the Cu_6_Sn_5_ solid-phase grains is:

$${{\varvec{C}}}_{{\varvec{r}}}={{\varvec{C}}}_{0}\mathbf{e}\mathbf{x}\mathbf{p}\left(\frac{2{\varvec{M}}{\varvec{\gamma}}}{{\varvec{R}}{\varvec{T}}{\varvec{\rho}}{\varvec{r}}}\right)$$

${{\varvec{C}}}_{{\varvec{r}}}$—The concentration of Cu atoms.

${{\varvec{C}}}_{0}$—Limiting solubility of solute atoms in Sn.

${\varvec{\gamma}}$—Specific surface energy of intermetallic compounds with Sn.

R is the gas constant, T is the temperature, ${\varvec{\rho}}$ is the density, M is the molar mass of Cu.

The copper atom source ratio (J1/J2) determines the size of r. When the radius r tends to infinity (i.e., loses its spherical shape):

$${{\varvec{C}}}_{{\varvec{r}}}={{\varvec{C}}}_{0}$$

Therefore, Unlike scalloped grains, the growth of faceted grains is predominantly influenced by the interfacial reaction flux. Specifically, when the intermetallic compound adopts a prismatic (faceted grain) morphology, the interfacial reaction process (J1) is dominated by the precipitation of additional Cu atoms from Sn. This aging process leads to the growth of Cu_6_Sn_5_ in a faceted grain manner along its length, rather than in a scalloped manner along the cross-section. Consequently, fast diffusion channels for copper atoms between grains remain intact and do not diminish as the compound grows. As a result, the decrease in chemical reaction flux is much smaller compared to scalloped grains.

Thus, When Co atoms are present in Sn, Cu atoms are more readily precipitated in Sn, and the copper atom source ratio (Cu atoms from Sn is J_1_; Cu atoms from intracrystalline diffusion within Cu_6_Sn_5_ is J_2_) determines the morphology of the intermetallic compounds. When additional Cu atoms precipitated from Sn dominate the interfacial reaction process, the intermetallic compounds grow in a prismatic (faceted grain) morphology.

**Figure 15:**
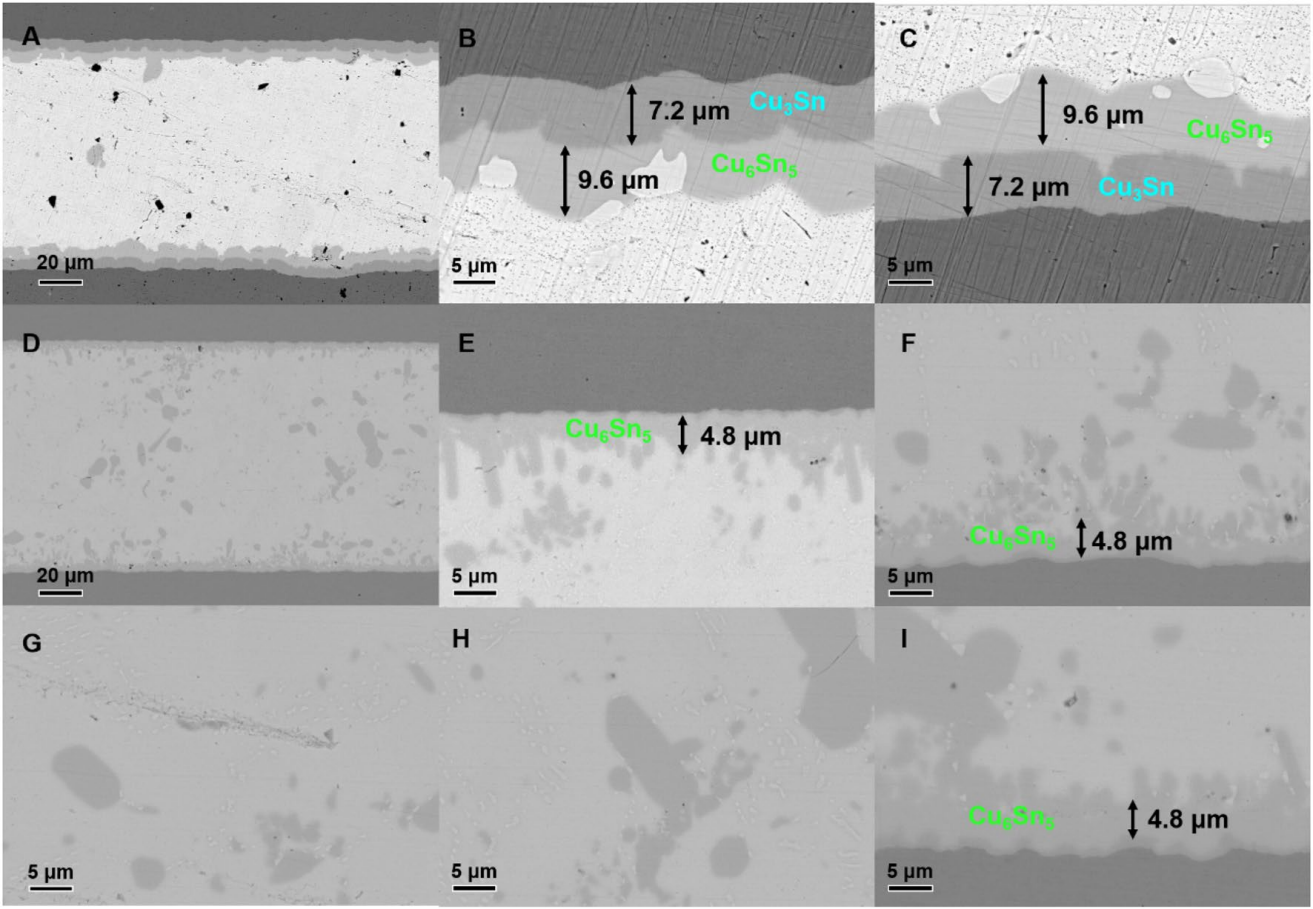
Electron microscopy of the joint interface after aging at 150 °C for 10 days (**A**) interface of the solder joint without **CoSn**_**3**_ (**B**) interface of the solder joint without **CoSn**_**3**_ (**C**) interface of the solder joint without **CoSn**_**3**_ (**D**–**I**) interface of the solder joint with 0.3 wt% **CoSn**_**3**_ Ns.

**Figure 16:**
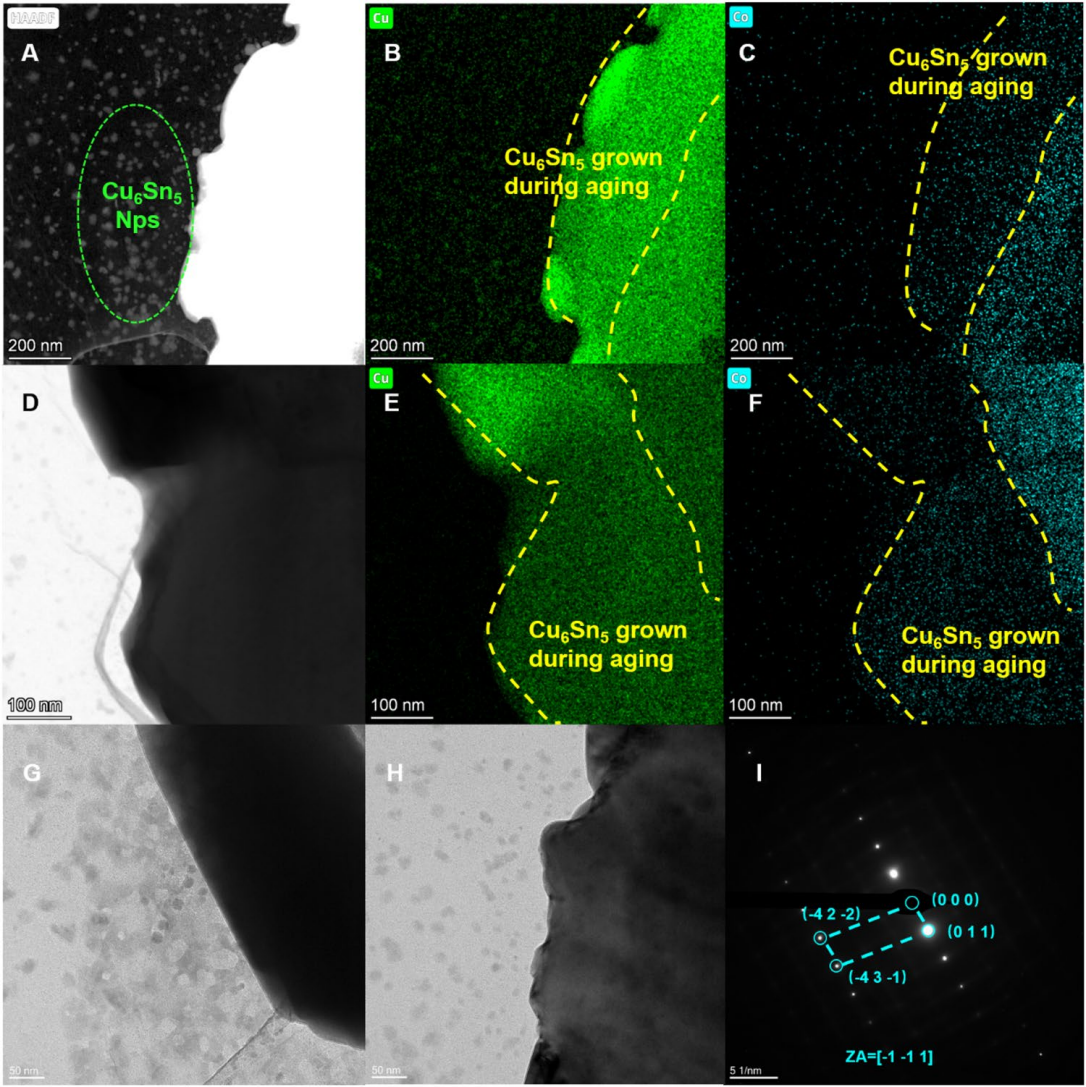
TEM images of the aged interface of the solder joint with 0.3 wt% **CoSn**_**3**_ Nps.

Surprisingly, scanning electron microscopy (SEM, Fig. 15) and transmission electron microscopy (TEM, Fig. 16) analysis of the aged samples revealed an unexpected outcome. After 10 days of aging, the intermetallic compounds in the **CoSn**_**3**_ nanoparticle-doped samples were significantly fewer compared to those in the samples without **CoSn**_**3**_ doping. Additionally, the Cu_6_Sn_5_ at the interface of the samples lacking **CoSn**_**3**_ doping exhibited continuous growth, which can adversely affect the mechanical properties of the joint. In contrast, the Cu_6_Sn_5_ in the **CoSn**_**3**_-doped joints exhibited an island-like shape (Figs. 15, 16) due to the presence of Co atoms in Sn, which reduced the solubility of Cu atoms and facilitated easier nucleation of Cu_6_Sn_5_. However, the faceted intermetallic compound morphology did not reduce the copper flux, as the Co element's presence inhibited the growth of Cu_6_Sn_5_. Instead, the Cu atoms preferred to nucleate new Cu_6_Sn_5_ rather than attaching to the existing Cu_6_Sn_5_ during the growth process.

Although **CoSn**_**3**_ nanoparticles tended to disappear after reflowing and were distributed in the Cu-Sn system solely in the form of Co elements, their presence enhanced the nucleation of Cu_6_Sn_5_ and led to the precipitation of numerous Cu_6_Sn_5_ nanoparticles in the interfacial system, effectively improving the joint's mechanical properties. Furthermore, the presence of elemental Co unexpectedly inhibited the development of Cu3Sn, resulting in a reduced number of Kirkendall voids during aging.

### The effect of **CoSn**_**3**_ on the mechanical properties

**Figure 17:**
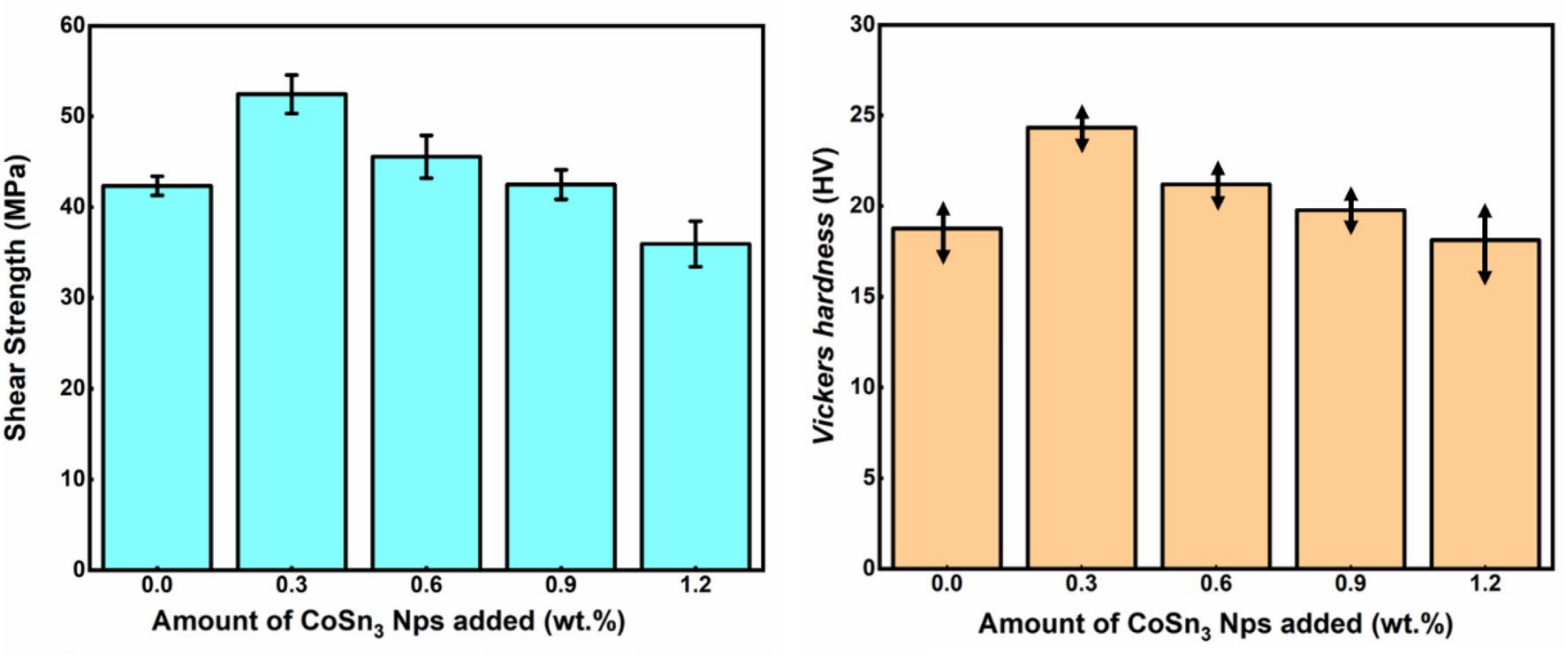
Mechanical properties of composite joints doped with different contents of **CoSn**_**3**_ Ns: Shear strength and Vickers hardness.

Our shear experiments conducted on joints doped with varying contents of **CoSn**_**3**_ provide supporting evidence for our earlier conclusions. Notably, the highest strength was observed in **CoSn**_**3**_ joints doped with a 0.3% mass ratio. The shear strength of these joints increased by 25% compared to undoped **CoSn**_**3**_ joints (Fig. 17). However, further increase in **CoSn**_**3**_ doping did not lead to higher strength. Instead, the fracture morphology indicated that the dissolution of more Co elements in Sn resulted in easier precipitation of Cu_6_Sn_5_, leading to the formation of aggregates of Cu_6_Sn_5_ within the joints, which negatively affected their mechanical properties.

**Figure 18:**
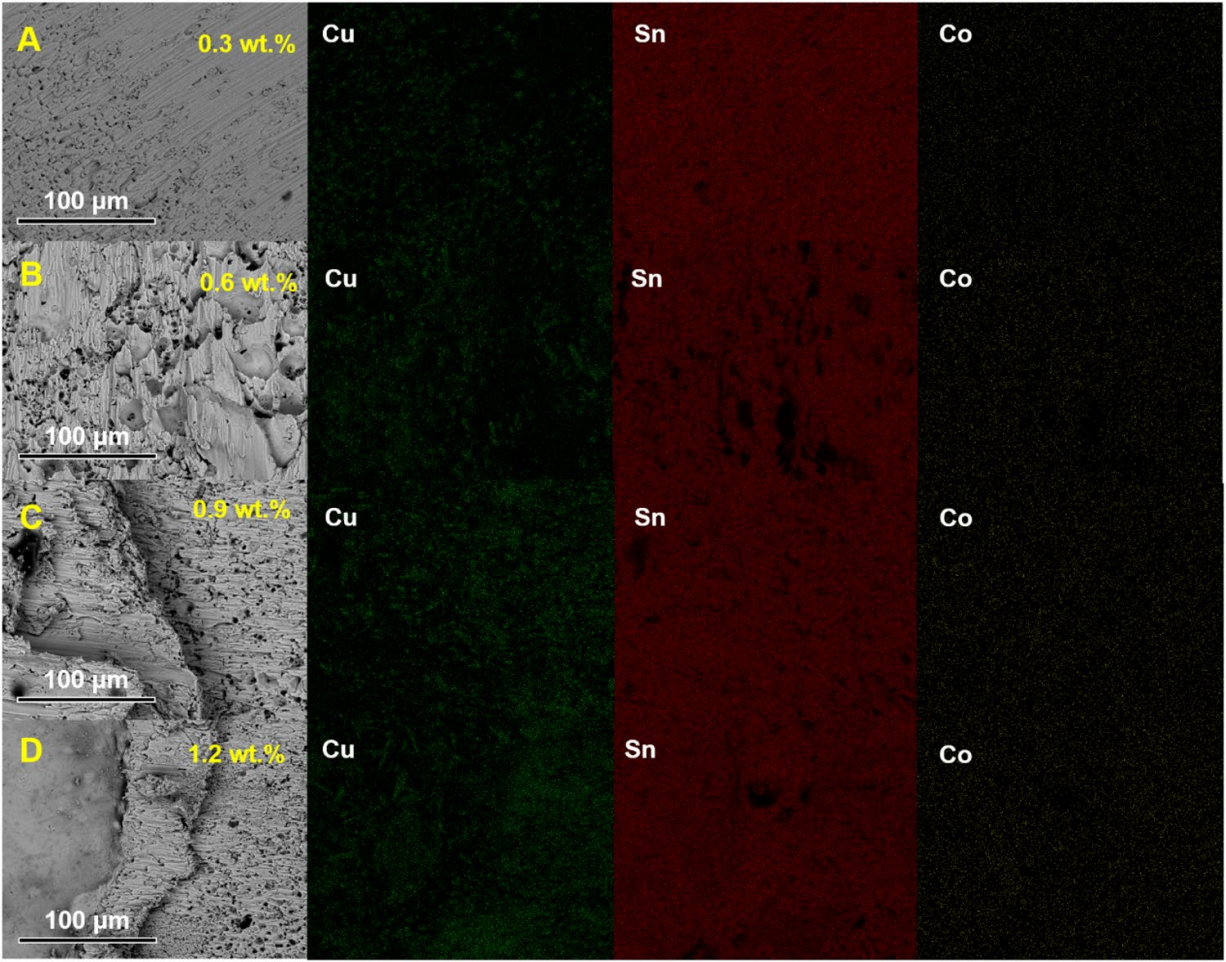
Elemental distributions in the fracture of composite joints doped with different contents of **CoSn**_**3**_ (**A**) 0.3 wt% (**B**) 0.6 wt% (**C**) 0.9 wt% (**D**) 1.2 wt%.

**Figure 19:**
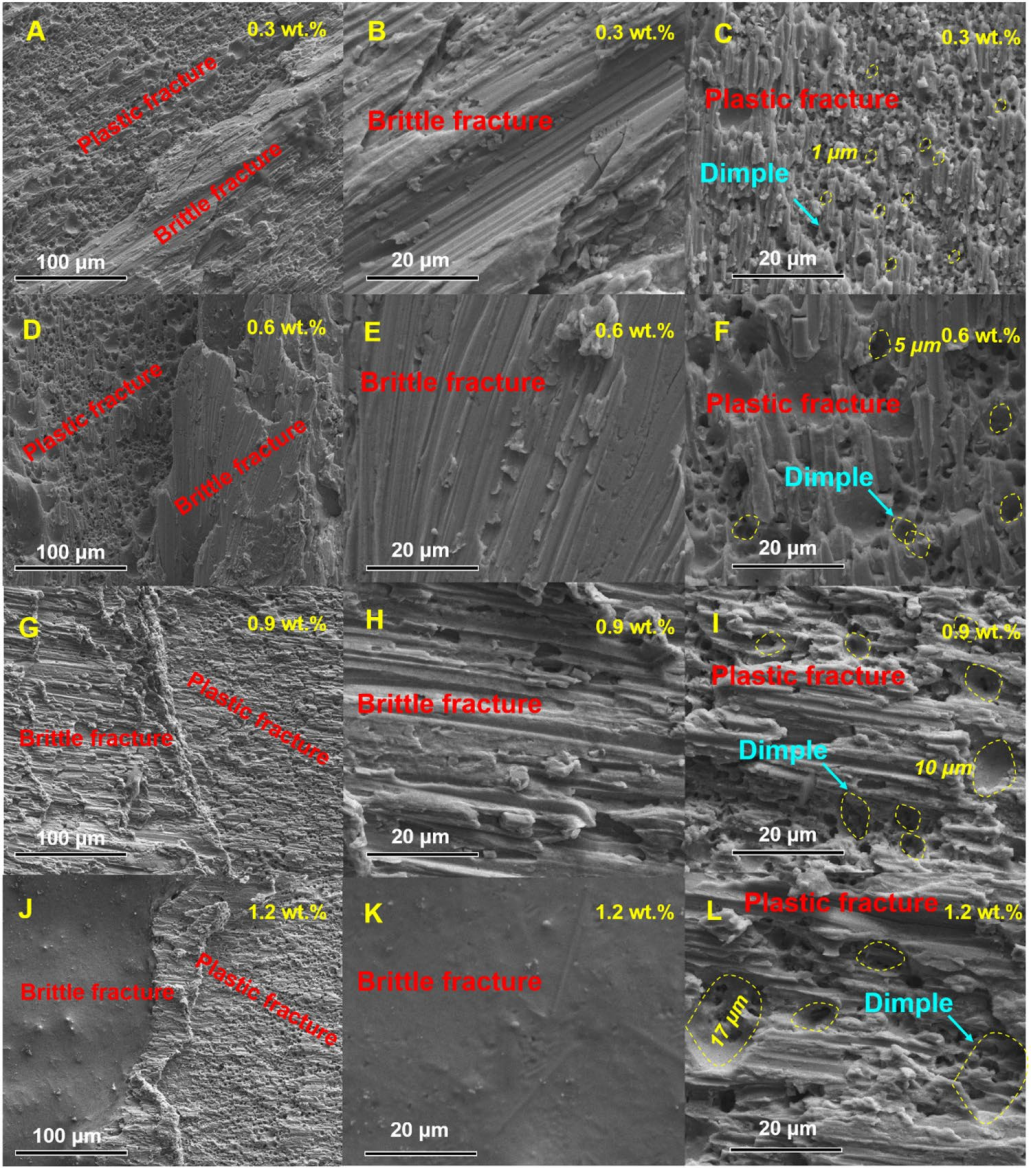
SEM images of composite joints doped with different contents of **CoSn**_**3**_ (**A**–**C**) 0.3 wt% (**D**–**F**) 0.6 wt% (**G**–**I**) 0.9 wt% (**J**–**L**) 1.2 wt%.

Notably, the EDX scans (Fig. 18) of the fracture interface did not reveal the presence of **CoSn**_**3**_ nanoparticles. Instead, the Co element was found to be uniformly distributed throughout the interface. However, a large number of Cu_6_Sn_5_ nanoparticles were detected at the interface (Fig. 19). This observation supports our earlier viewpoint that **CoSn**_**3**_ dissolves in Sn, causing a reduction in the solubility of Cu atoms in Sn, and subsequently leading to the formation of numerous Cu_6_Sn_5_ nanoparticles.

The shear experiments and fracture morphology analysis underscore the significance of carefully controlling the **CoSn**_**3**_ doping level to achieve optimal mechanical properties in solder joints. An appropriate mass ratio of **CoSn**_**3**_ enhances joint strength, but excessive **CoSn**_**3**_ doping may result in the formation of Cu_6_Sn_5_ aggregates, negatively impacting the joint's performance.

The introduction of appropriate Co elements into the Sn matrix has been found to effectively stimulate the nucleation and uniform distribution of Cu_6_Sn_5_ nanoparticles. This phenomenon was further associated with the microstructure's influence on the mechanical properties, as observed through nanoindentation analyses. The experimental results demonstrated a notable enhancement in hardness, modulus, and creep resistance for joints doped with **CoSn**_**3**_. The distribution of Cu_6_Sn_5_ nanoparticles contributed to the refinement of the eutectic microstructure and concurrently impeded grain boundary movement, thereby leading to improved tensile properties. The presence of finer and dispersed intermetallic compounds also hindered dislocation movement and effectively stabilized grain boundaries, resulting in enhanced creep resistance through diffusion strengthening.

## Conclusion

Sn3Ag plating is a widely used electronic packaging material, and we have developed a novel composite plating method involving CoSn_3_ nanocrystals doped with SnAg plating. Through this method, we successfully deposited CoSn_3_ nanocrystals within the Sn3Ag plating layer on a Cu substrate. After reflow, the CoSn_3_-SnAg composite joints exhibited several unique properties:

CoSn_3_ nanocrystals dissolved in Sn during the reflow process at 250 °C.

The Co element participated in the Cu-Sn reaction, displacing some Cu atoms to form (Cu,Co)_6_Sn_5_. The EDX results revealed a highly coincident distribution of Cu and Co elements in the reflowed joints.

The dissolution of Co atoms in Sn led to a decrease in the solubility of Cu atoms in Sn, promoting the nucleation of **Cu**_**6**_**Sn**_**5**_. As a result, **Cu**_**6**_**Sn**_**5**_ nanoparticles were abundantly distributed in the joints.

The presence of dissolved Co atoms in Sn reduced the degree of supercooling required for Sn nucleation, enabling Sn to grow in six-fold cyclic twins at a lower degree of supercooling.

In this joint, **Cu**_**6**_**Sn**_**5**_ exhibited a faceted growth pattern, which was attributed to the Co atoms in Sn preventing Cu atoms from dissolving in Sn. As a result, grain boundary diffusion dominated the growth of **Cu**_**6**_**Sn**_**5**_, influenced by the interfacial reaction flux.

The presence of **Cu**_**6**_**Sn**_**5**_ nanoparticles within the joints exerted a pinning effect on dislocations and grain boundaries, effectively improving the mechanical properties of the joints.

Overall, the CoSn_3_-SnAg composite joints displayed a combination of unique features resulting from the interaction of CoSn_3_ nanocrystals and SnAg plating, which contributed to enhanced mechanical properties and overall performance in electronic packaging applications.

## Data Availability

All data generated or analyzed during this study are included in this published article [and its supplementary information files.
